# Hereditary breast and ovarian cancer: assessment of point mutations and copy number variations in Brazilian patients

**DOI:** 10.1186/1471-2350-15-55

**Published:** 2014-05-15

**Authors:** Felipe C Silva, Bianca CG Lisboa, Marcia CP Figueiredo, Giovana T Torrezan, Érika MM Santos, Ana C Krepischi, Benedito M Rossi, Maria I Achatz, Dirce M Carraro

**Affiliations:** 1 Laboratory of Genomics and Molecular Biology, CIPE - A. C. Camargo Cancer Center, São Paulo, Brazil; 2 Department of Colorectal Tumors, A. C. Camargo Cancer Center, São Paulo, Brazil; 3 National Institute of Science and Technology in Oncogenomics (INCITO), São Paulo, Brazil; 4 Department of Oncogenetics, A. C. Camargo Cancer Center, São Paulo, Brazil

**Keywords:** Breast cancer, Mutation, *BRCA1*, *BRCA2*, *HBOC*, *CHEK 1100delC*, *TP53 R337H*

## Abstract

**Background:**

Germ line mutations in *BRCA1* and *BRCA2* (*BRCA1/2*) and other susceptibility genes have been identified as genetic causes of hereditary breast and ovarian cancer (HBOC). To identify the disease-causing mutations in a cohort of 120 Brazilian women fulfilling criteria for HBOC, we carried out a comprehensive screening of *BRCA1/2*, *TP53* R337H, *CHEK2* 1100delC, followed by an analysis of copy number variations in 14 additional breast cancer susceptibility genes (*PTEN*, *ATM*, *NBN*, *RAD50*, *RAD51*, *BRIP1*, *PALB2*, *MLH1*, *MSH2*, *MSH6*, *TP53*, *CDKN2A*, *CDH1* and *CTNNB1*).

**Methods:**

Capillary sequencing and multiplex ligation-dependent probe amplification (MLPA) were used for detecting point mutations and copy number variations (CNVs), respectively, for the *BRCA1* and *BRCA2* genes; capillary sequencing was used for point mutation for both variants *TP53* R337H and *CHEK2* 1100delC, and finally array comparative genomic hybridization (array-CGH) was used for identifying CNVs in the 14 additional genes.

**Results:**

The positive detection rate in our series was 26%. *BRCA1* pathogenic mutations were found in 20 cases, including two cases with CNVs, whereas *BRCA2* mutations were found in 7 cases. We also found three patients with the *TP53* R337H mutation and one patient with the *CHEK2* 1100delC mutation. Seven (25%) pathogenic mutations in *BRCA1/2* were firstly described, including a splice-site *BRCA1* mutation for which pathogenicity was confirmed by the presence of an aberrant transcript showing the loss of the last 62 bp of exon 7. Microdeletions of exon 4 in *ATM* and exon 2 in *PTEN* were identified in *BRCA2*-mutated and *BRCA1/2*-negative patients, respectively.

**Conclusions:**

In summary, our results showed a high frequency of *BRCA1/2* mutations and a higher prevalence of *BRCA1* (64.5%) gene. Moreover, the detection of the *TP53* R337H variant in our series and the fact that this variant has a founder effect in our population prompted us to suggest that all female breast cancer patients with clinical criteria for HBOC and negative for *BRCA1/2* genes should be tested for the *TP53* R337H variant. Furthermore, the presence of genomic structural rearrangement resulting in CNVs in other genes that predispose breast cancer in conjunction with *BRCA2* point mutations demonstrated a highly complex genetic etiology in Brazilian breast cancer families.

## Background

Hereditary breast and ovarian cancer (HBOC) accounts for 5-10% of all breast cancer (BC) cases and is inherited in an autosomal dominant fashion. Nearly 30% of HBOC patients harbor germ line point mutations or genomic structural rearrangements that result in copy number variations (CNVs) in *BRCA1*/*2* genes*,* with a lifetime risk of 45-70% for BC and 20-40% for ovarian cancer [1]. Mutations in these genes also confer a slightly increased risk of other types of cancer, such as pancreatic, primary peritoneal, prostate, male breast and fallopian tube cancer [2].

Germ line *BRCA1* (MIM# 113705) and *BRCA2* (MIM# 600185) mutations are also frequently found in isolated cases of bilateral and/or early-onset BC [3]. The frequency of these mutations is variable because a higher frequency of *BRCA1* mutations has been described in the United States [4], whereas a clear prevalence of *BRCA2* mutations has been reported in Icelandic BC families [5]. Conversely, similar mutation frequency in both genes has been described in French Canadian and British families [6,7].

Inherited mutations in other genes also influence the risk of BC. The *CHEK2* 1100delC has been associated with higher BC risks [8], conferring a two-fold increase in BC risk for women and a ten-fold increase for men [8-10]. Recent studies in cancer-prone families in southeast Brazil have identified a founder germ line *TP53* mutation (p.R337H) at a higher prevalence (1:3,000) than the others germ line *TP53* mutations [11]. A variety of cancer types have been found in *TP53* p.R337H-carrying families, such as soft tissue sarcomas, brain tumors, adrenocortical carcinomas and breast cancers [11].

Screening for mutations in the tumor suppressor genes *BRCA1* and *BRCA2* is of great significance for breast and ovarian cancer prevention and early detection. When a mutation is identified, the cancer risk can be reduced via prophylactic mastectomy; patients can also seek effective screening strategies to detect breast cancer earlier [12]. Moreover, whether a mutation is detected, the genetic testing can be extended to relatives who can enter in specific screening programs for carriers or follow the strategy for the general population (non-carriers). More recently, emerging therapies, such as PARP inhibitors in combination with conventional treatment, have been shown to be more effective for *BRCA1* and *BRCA2* mutation carriers [13].

The complex genetic basis of hereditary breast cancer prompted us to perform a comprehensive genetic investigation of 120 Brazilian patients fulfilling clinical criteria for HBOC. We screened the *BRCA1/2* genes for point mutations and CNVs. We also evaluated the presence of the *CHEK2* 1100delC and *TP53* R337H variants via sequencing and used array comparative genomic hybridization (array-CGH) to investigate CNVs in 14 additional breast cancer-predisposing genes: *PTEN*, *ATM*, *NBN*, *RAD50*, *RAD51*, *BRIP1*, *PALB2*, *MLH1*, *MSH2*, *MSH6*, *TP53*, *CDKN2A*, *CDH1* and *CTNNB1*.

## Methods

### Patients

One hundred twenty unrelated breast cancer patients fulfilling criteria for hereditary breast and ovarian cancer (HBOC) were recruited from 2007 to 2010 for this study. The inclusion criteria were: 1) Breast cancer diagnosed ≤ 45 years of age (no family history); 2) Breast cancer diagnosed ≤ 45 years of age with 1 or more close blood relative with breast/ovarian/fallopian tube/primary peritoneal cancer at any age; 3) Breast cancer diagnosed <45 ≤ 50 years of age with 1 or more blood relative with breast/ovarian/fallopian tube/primary peritoneal cancer ≤ 50 years of age; 4) Breast cancer diagnosed >50 of age with 1 or more blood relative with breast/ovarian/fallopian tube/primary peritoneal cancer at any age; 5) Two primary BC when the first occurrence was prior to age 50; 6) Breast cancer with a history of ovarian/ fallopian tube/primary peritoneal cancer at any age; 7) For an individual with an ethnicity that is associated with a higher mutation frequency (e.g., Ashkenazi Jewish); 8) Personal history of ovarian/fallopian tube/primary peritoneal cancer; 9) Personal history of male breast cancer. All enrolled individuals received genetic counseling and signed an informed consent. This study was performed in compliance with the Helsinki Declaration and was approved by the ethics committee of the A C Camargo Cancer Center (approval number: 870/06-B). The complete clinical and molecular information of the patients is given in the Additional file 1.

### Point mutation screening

DNA from peripheral blood was purified using the Puregene Genomic DNA Isolation kit (Quiagen, Hilden, Germany) according to manufacturer’s instructions. The entire coding sequence and exon-intron boundaries of the *BRCA1* (U14680 or NM_007294.3) and *BRCA2* (U43746 or NM_000059.1) genes were evaluated. The *CHEK2* gene (NM_007194.3) and the *TP53* gene (NM_000546.5) were screened solely for the c.1100delC and p.R337H mutations, respectively. All PCR products were sequenced in both forward and reverse directions on an ABI Prism 3130xl genetic analyzer (Life Technologies, Foster City, USA). Mutations were recorded and referenced with respect to the cDNA sequence using the nomenclature proposed by the BIC database [14]. PCR conditions and primer sequences are available upon request.

### Investigation of *BRCA1/2* CNVs by MLPA

Patients negative for *BRCA1/2* mutations were investigated for CNVs in these genes. Exonic deletions and duplications affecting *BRCA1* and *BRCA2* genes were investigated on genomic DNA using the multiplex ligation-dependent probe amplification (MLPA) commercial kits P087-B1 and P045-B3 (MRC-Holland, Amsterdam, The Netherlands) according to the manufacturer’s recommendations.

### Classification of variants

The BIC database was searched for all *BRCA1* and *BRCA2* alterations [14]. Unreported mutations that generated a premature stop codon (nonsense and frameshift) were classified as pathogenic. Missense alterations classified as class 3 in the IARC_LOVD database [15] or unknown in the BIC database were considered to be variants of uncertain significance (VUSs). Variants classified as class 1 and 2 using the IARC_LOVD were considered to be wild type. In cases of inconsistency between the databases, the classification from IARC-LOVD prevailed. Additionally, the VUSs were characterized using three *in silico* protein prediction algorithms: SIFT [16], POLYPHEN-2 [17] and Align-GVGD [18].

### Transcript analysis

For transcriptional analysis of the novel splice site variant, frozen tumor tissues were obtained from the carrier and from a sporadic breast tumor that was negative for mutations in the *BRCA1* gene, which was used as a control sample. RNA samples were purified using the Precellys 24® equipment (Carlsbad, California, USA), followed by total RNA extraction using an RNeasy Mini kit (Qiagen, Venlo, The Netherlands). The first strand cDNA was synthesized from 2 μg of total RNA using a random hexamer primer with the Superscript first strand system for RT-PCR (Life Technologies, Foster City, USA). RT-PCR fragments of the index patient and the control sample were both obtained according to standard PCR protocols using primers adjacent to the exon involved in the splice site mutation. All RT-PCR products were inserted into the T/A plasmid vector pTZ57R/T using the InsT/Aclone PCR Product Cloning Kit (Thermo Fisher Scientific, USA), and the ligated plasmid was used for transformation in DH10B *E. coli* cells via electroporation (2.5 KV, 25 μ FD, 200 OHMS), followed by single-colony sequencing on the ABI 3130xl sequencer using M13 primers.

### Screening of copy number alterations via array comparative genomic hybridization (array-CGH)

Genomic CNVs affecting 14 cancer susceptibility genes (*PTEN*, *TP53*, *ATM*, *NBN*, *RAD50*, *BRIP1*, *PALB2*, *RAD51*, *MLH1*, *MSH2*, *MSH6*, *CDKN2A*, *CDH1* and *CTNNB1*) were investigated in oligo-based array-CGH data obtained from a previous study [19]. All hybridizations were gender-matched and processed in reverse labeling duplicates; experiments were carried out using the 180 K whole genome platform (design 22060, Agilent Technologies, Santa Clara, CA, USA). The Agilent Genomic Workbench software was used for the detection of CNVs (deletion and duplications) with the aberration detection method 2 (ADM-2) and a threshold of 6.7. The duplication or deletion of genomic segments were declared when one probe exhibited a log_2_ ratio of Cy3/Cy5 > 0.70 or < -0.70, respectively. A careful visual inspection was performed to filter out poor quality hybridizations and noisy data. Additionally, alterations located more than 3 kb upstream or downstream of the coding exons were excluded. The Database of Genomic Variants (DGV-HG19, http://projects.tcag.ca/variation/) was used to exclude common variants detected in the general population. Only alterations detected in both experiments of the same patient were considered.

### Gene dosage qPCR

To validate the genomic DNA CNVs, we used the quantitative duplex PCR method previously described [20]. *GAPDH* was used as a reference gene. For array-CGH probes located in introns, PCR primers were designed to encompass the closest exon.

## Results

### Screening of *BRCA1/2*: point mutations and copy number variations; *CHEK2* 1100delC and *TP53* R337H

The mean age of diagnosis of the first primary tumor in all 120 patients was 43 years-old (yo), with a range from 22 to 88. Thirty-one out of 120 patients (26%) were found to harbor pathogenic mutations, including 20 for *BRCA1* (64.5%), seven for *BRCA2* (22.5%), three for *TP53* R337H (10%) and one for *CHEK2* 1100delC (3%). In the *BRCA1* gene, 20 patients presented 18 different mutations, of which 16 were point mutations (89%) (Six nonsense, six frameshift, two splice site and two missense) and two were CNVs, one deletion encompassing exons 16 and 17 and a rare case of exon 24 amplification detected within *BRCA1* (11%) (Table 1 and Additional file 2). The Ashkenazi Jewish mutation c.5382insC was the most recurrent and was found in three cases. The seven *BRCA2* carriers presented six distinct mutations, of which three were nonsense and three were frameshift. The nonsense variant c.9709A > T was found in two cases. No CNVs were detected in *BRCA2*. Moreover, seven out of 28 (25%) *BRCA1/2*-carriers were found to harbor novel pathogenic mutations, five in *BRCA1* and two in *BRCA2*.

**Table 1:** **Clinical and molecular description of the *BRCA1*, *BRCA2, CHEK2 *** **1100delC and *TP53 *** **R337H mutation carriers**

**Proband**	**Age of onset**	**Tumor**	**Clinical criteria**	**Gene**	**Alteration**	**Mutation type**	**Exon**	**Reference**
**SM-01**	**39**	Breast	HBOC	*BRCA1*	c.5203delTT	Frameshift	18	BIC
**SM-17**	**49/51**	Ovarian/Breast	HBOC	*BRCA1*	c.3376 T > G; p.L1086X	Nonsense	11	BIC
**SM-25**	**69**	Breast	HBOC	*BRCA1*	c.120A > G; p.M1V	Missense	2	BIC
**SM-69**	**33**	Breast	HBOC	*BRCA1*	c.5242C > A; p.A1708E	Missense	18	BIC
**SM-74**	**50**	Hemangioblastoma	HBOC	*BRCA1*	c.2080delA	Frameshift	11	BIC
**MO-07**	**34**	Breast	HBOC	*BRCA1*	c.5382insC	Frameshift	20	BIC
**MO-09**	**42**	Breast	HBOC	*BRCA1*	c.5382insC	Frameshift	20	BIC
**MO-45**	**48**	Breast	HBOC	*BRCA1*	c.5382insC	Frameshift	20	BIC
**MO-13**	**32/44**	Breast/Skin	HBOC	*BRCA1*	c.4831del5	Frameshift	16	BIC
**MO-26**	**37**	Breast	HBOC	*BRCA1*	c.1499insA	Frameshift	11	BIC
**MO-31**	**38/63**	Breast/Peritoneal	HBOC	*BRCA1*	c.5563G > A; p.W1815X	Nonsense	23	BIC
**MO-37**	**(-)**	Breast	HBOC	*BRCA1*	c.3759G > T; p.E1214X	Nonsense	11	BIC
**MO-38**	**42/44**	Breast/Breast	HBOC	*BRCA1*	c.307 T > A; p.L63X	Nonsense	5	BIC
**SM-50**	**36/47**	Breast/Breast	HBOC	*BRCA1*	c.4794 + 1G > A	Splice site	15	BIC
**SM-80**	**42/48**	Breast/Ovarian	HBOC	*BRCA1*	c.1446 A > T; p.K443X	Nonsense	11	Current study
**SM-81**	**55**	Fallopian Tube	HBOC	*BRCA1*	c.5582insT	Frameshift	23	Current study
**SM-89**	**40**	Breast	HBOC	*BRCA1*	c.4406 C > A; p.Y1429X	Nonsense	13	Current study
**MO-15**	**43/56**	Breast/Ovarian	HBOC	*BRCA1*	c.560 + 2 T > A	Splice site	7	Carraro et al. [28]
**M0-28**	**48**	Breast	HBOC	*BRCA1*	Exon 24 amplification	LGR	24	Current study
**SM-03**	**36/36**	Breast/Ovarian	HBOC	*BRCA1*	Exon 16–17 deletion	LGR	16-17	BIC
**SM-08**	**33**	Breast	HBOC	*BRCA2*	c.6174delT	Frameshift	11	BIC
**SM-46**	**32**	Breast	HBOC	*BRCA2*	c.9709 A > T; p.K3161X	Nonsense	25	BIC
**SM-61**	**51**	Breast	HBOC	*BRCA2*	c.9709A > T; p.K3161X	Nonsense	25	BIC
**SM-104**	**32**	Breast	HBOC	*BRCA2*	c.3034del4	Frameshift	11	BIC
**MO-02**	**53/63**	Breast/Breast	HBOC	*BRCA2*	c.9610C > T, p.R3128X	Nonsense	25	BIC
**SM-53**	**30/49**	Breast/Thyroid	HBOC	*BRCA2*	c.6242del4	Frameshift	11	Current study
**SM-84**	**35**	Breast	HBOC	*BRCA2*	c.8423 T > G; p.L2732X	Nonsense	18	Current study
**MO-41**	**47**	Breast	HBOC	*CHEK2*	c.1100delC; p.Thr367MetfsX15	Frameshift	10	BIC
**SM-31**	**49**	Breast	HBOC	*TP53*	c.1010G > A; p.R337H	Missense	10	IARC TP53
**SM-31**	**49**	Breast	HBOC	*TP53*	c.1010G > A; p.R337H	Missense	10	IARC TP53
**SM-82**	**29**	Breast	HBOC	*TP53*	c.1010G > A; p.R337H	Missense	10	IARC TP53

(-) Not available; LGR: Large Genomic Rearrangement; BIC: Breast cancer information core database; IARC TP53: IARC TP53 database.

**Figure 1:**
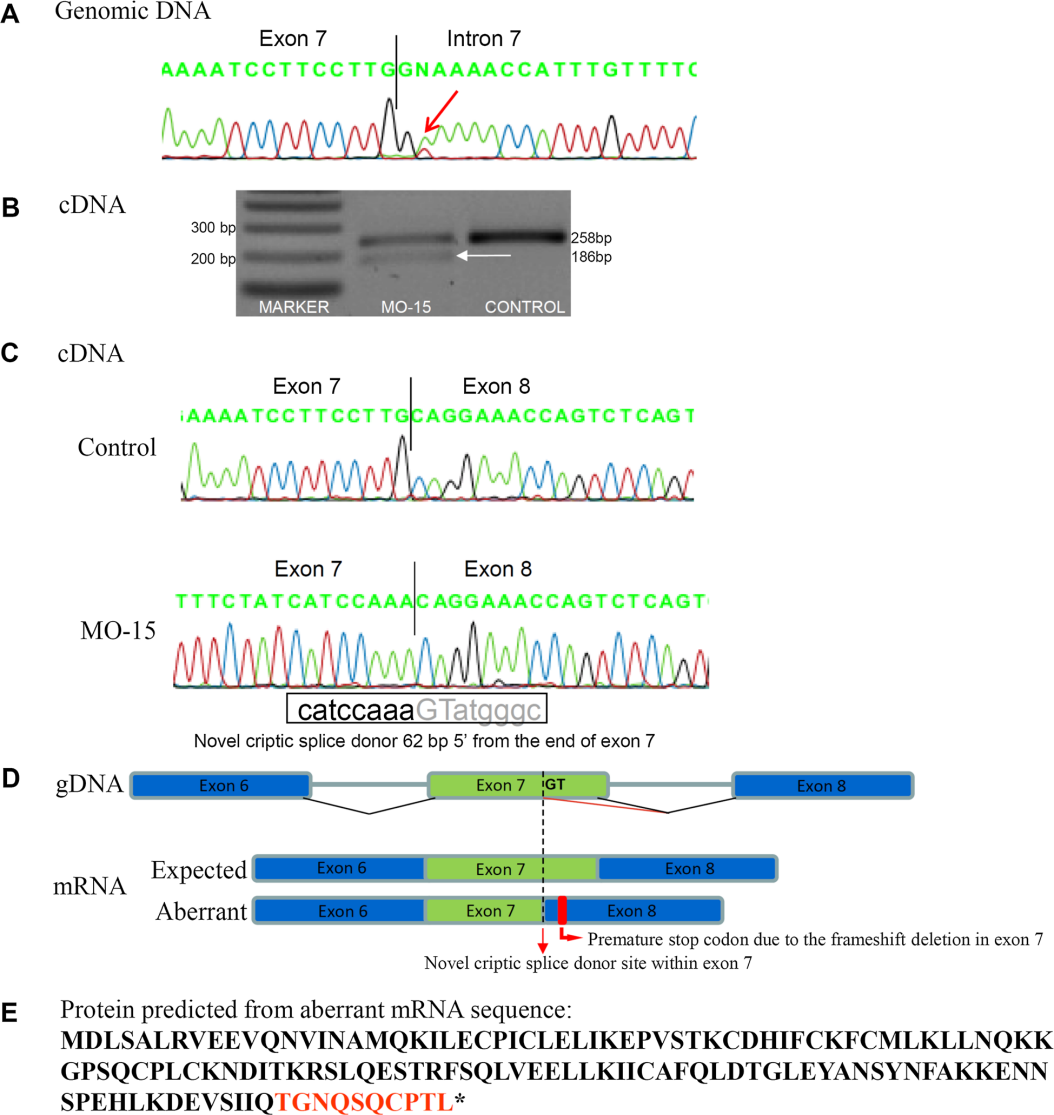
**Characterization of a novel *BRCA1 *splice site variant (c.560 + 2 T > A) in one HBOC patient. A**: Sequencing pattern of *BRCA1* exon 7 from blood cell genomic DNA showing the c.560 + 2 T > A mutation. **B**: Agarose gel showing RT-PCR products obtained from the cDNA of a tumor from patient MO-15 and one control sample (sporadic tumor negative for the c.560 + 2 T > A) using a forward primer in exon 6 and a reverse primer in exon 8 of *BRCA1*. An additional 186-bp cDNA fragment caused by the partial deletion of exon 7 was observed in the tumor sample of patient MO-15. **C**: Above, partial sequence of the expected fragment (258 bp) in the control tumor sample showing the exon 7–8 junction; below, partial sequence of the patient tumor cDNA showing the creation of a novel cryptic splice donor site causing the deletion of the last 62 bp of *BRCA1* exon 7 in the aberrant transcript. **D**: Schematic representation of the premature stop codon (p.Ser127Thrfs*11) created in the *BRCA1* mRNA after the frameshift deletion of the last 62 bp of exon 7 caused by the germ line splice site variant c.560 + 2 T > A. **E**: Amino acid sequence of the expected truncated protein (137 aa) showing the alteration of 10 amino acids (in red) and creation of a premature stop codon (*).

Interestingly, patient MO-15 was found to harbor a germ line splice site mutation (c.560 + 2 T > A) in intron 7 of the *BRCA1* gene (Figure 1A). RT-PCR products encompassing part of exon 6 to exon 8 revealed the presence of a 186-bp fragment in addition to the expected fragment of 258 bp (Figure 1B). Sequencing of the 186-bp fragment disclosed a frameshift deletion of the last 62 bp of exon 7 due to the activation of a novel cryptic splice site within exon 7 (Figure 1C). Figure 1D shows the schematic representation of the premature stop codon created in the mRNA after the deletion of 62 bp of exon 7 (r.[=, 499_560del); p.Ser127Thrfs*11) caused by the germ line splice site mutation c.560 + 2 T > A. The predicted protein from the aberrant mRNA apparently created an isoform of 137aa (Figure 1E).

**Figure 2:**
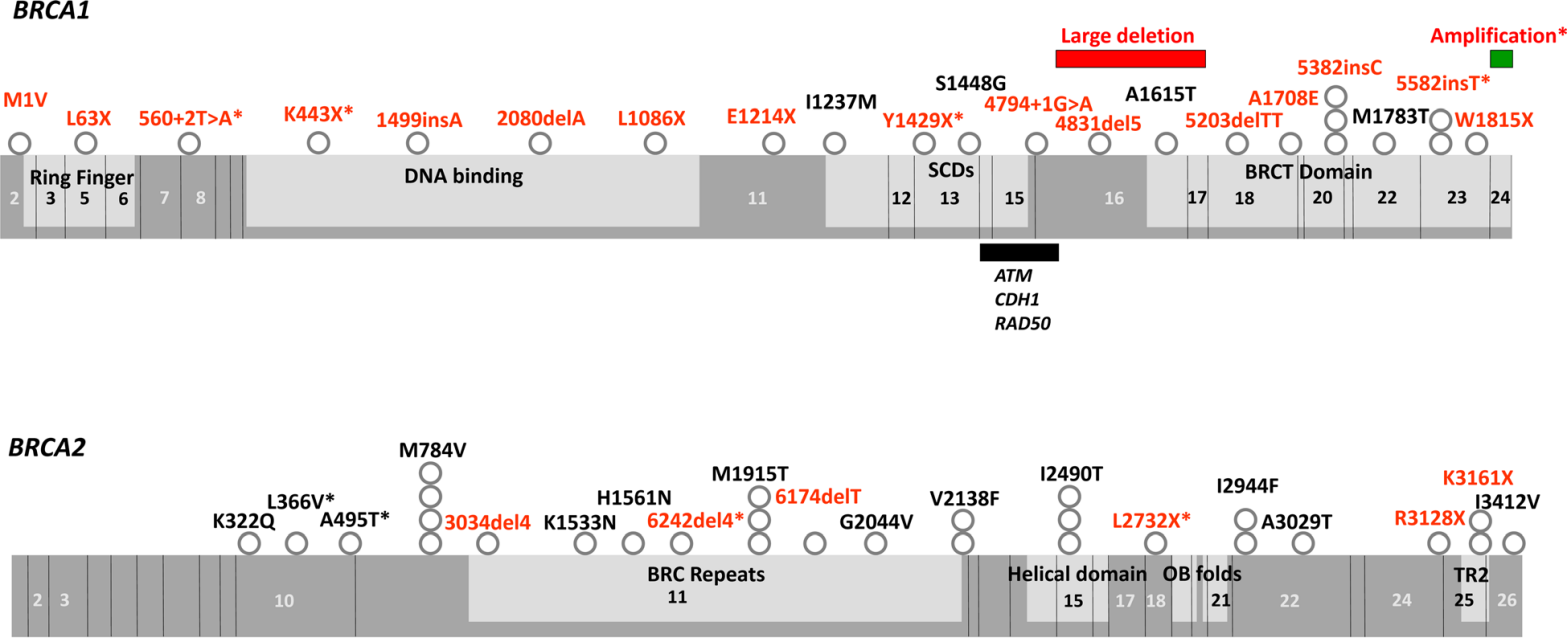
**Schematic representations of *BRCA1/2 *proteins.***BRCA1/2* proteins with their functional domains and the localization of all identified pathogenic mutations (red labels) and VUSs (black labels). Novel alterations are marked with an asterisk. The frequency of each alteration is represented by gray dots. The red and green bars represent the detected genomic rearrangements. The black bar represents the *ATM*, *CDH1* and *RAD50* binding domain.

Nineteen out of 120 HBOC patients (16%) harbored VUSs according to our criteria (based on BIC and/or IARC-LOVD databases - see the Methods section). Among the VUS carriers, 17 variants were distinct, and two of them were described for the first time (Table 2). The VUS evaluation using the three protein prediction algorithms (PolyPhen, SIFT and GVGD-Align) showed that six were classified as likely pathogenic by at least one algorithm (three in one algorithm, two in two algorithms and one in all three algorithms). The schematic representation of all VUSs in the *BRCA1* and *BRCA2* genes and their functional domains on the protein are shown in Figure 2.

**Table 2:** Variants of uncertain significance (VUSs)

**Variant**	**No. of families carrying VUS**	**Co-occurrence with pathogenic mutation**	**Gene**	**BIC**	**In silico analysis**			**IARC-LOVD database**
					**Polyphen-2**	**SIFT**	**Align GVGD**	
p.I1237M	1	No	*BRCA1*	Unknown	Benign	Tolerated	C0	-
p.S1448G	1	No	*BRCA1*	Unknown	Benign	Affect	C0	-
p.A1615T	1	No	*BRCA1*	Unknown	Possibly	Affect	C0	-
p.M1783T	1	No	*BRCA1*	Unknown	Probably	Affect	C55	-
p.K322Q	1	No	*BRCA2*	Unknown	Benign	Affect	C0	-
p.L366V	1	No	*BRCA2*	Novel	Benign	Tolerated	C0	-
p.A495T	1	No	*BRCA2*	Novel	Benign	Tolerated	C0	-
p.M784V	4	No	*BRCA2*	Unknown	Benign	Tolerated	C0	Class 3
p.K1533N	1	No	*BRCA2*	Unknown	Benign	Tolerated	C0	-
p.H1561N	1	Yes	*BRCA2*	Unknown	Benign	Tolerated	C0	-
p.M1915T	3	No	*BRCA2*	Unknown	Benign	Tolerated	C0	-
p.G2044V	1	Yes	*BRCA2*	Unknown	Benign	Tolerated	C0	-
p.V2138F	2	Yes	*BRCA2*	Unknown	Benign	Tolerated	C0	-
p.I2490T	3	Yes	*BRCA2*	Unknown	Benign	Tolerated	C45	-
p.I2944F	2	Yes	*BRCA2*	Unknown	Probably	Affect	C0	-
p.A3029T	1	No	*BRCA2*	Unknown	Benign	Tolerated	C0	-
p.I3412V	1	Yes	*BRCA2*	Unknown	Benign	Tolerated	C0	

Align GVGD: C0 (Less likely to interfere in protein function), C15, C25, C35, C45, C55, C65 (More likely to interfere in protein function); Polyphen: Variant Benign, Possibly damaging and Probably damaging; SIFT: Variant Tolerated (benign) or Affect protein function. LOVD-IARC: class 1 (Not pathogenic or of no clinical significance), class 2 (Likely not pathogenic or of little clinical), class 3 (Uncertain), class 4 (Likely pathogenic), class 5 (definitely pathogenic).

### Mutation frequency by inclusion criteria

**Figure 3:**
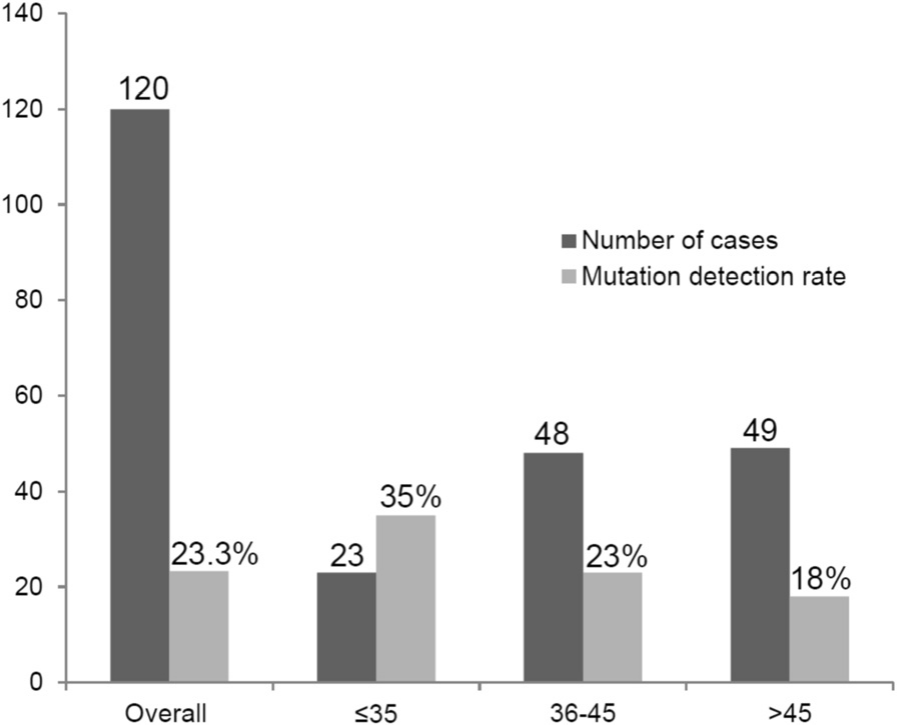
***BRCA1/2 *mutation frequency.** Mutation frequency according to age of cancer onset.

Overall, the mutation detection rate was 26%; however, when selecting according to age of cancer onset, young women (≤35 yo) had the highest rate at 35% (Figure 3). Regarding specific HBOC criteria fulfilled by each family, the majority of mutation carriers (45%) met criterion 2 *(Breast cancer diagnosed ≤ 45 yo and familial history positive for BC)*. Four out of five patients fulfilling the criterion for Ashkenazi Jewish ancestry (criterion 7) harbored pathogenic mutations, and patients fulfilling criterion 6 showed a detection rate of 44% (4/9). Patients younger than 45 yo without a family history of cancer (criterion 1) did not present pathogenic mutations in our cohort (Table 3).

**Table 3:** Mutation detection rate according to inclusion criteria

**Hereditary Breast and Ovarian Cancer (HBOC)**	**Distribution by criterion**	**Number of mutation carriers**	**Positive detection rate**
1. BC ≤ 45 years of age (no family history)	11	0	0%
2. BC ≤ 45 years of age with ≥ 1 relative with BC, OC, FTC and PPC at any age	42	14	33%
3. BC < 45 ≤ 50 years of age with ≥ 1 relative with BC, OC, FTC and PPC ≤ 50 years of age	16	2	12%
4. BC >50 years of age with ≥ 1 relatives with BC, OC, FTC and PPC at any age	28	5	18%
5. Two BC when the first occurrence was prior to age 50	5	1	20%
6. BC at any age plus OC, FTC and PPC at any age	9	4	44%
7. Ashkenazi Jewish ancestry	5	4	80%
8. OC, FTC and PPC at any age	3	1	33%
9. Male BC	1	0	0%

BC: Breast cancer; OC: ovarian cancer; FTC: Fallopian tube cancer; PPC: Primary peritoneal cancer.

### Array CGH and gene dosage qPCR

**Figure 4:**
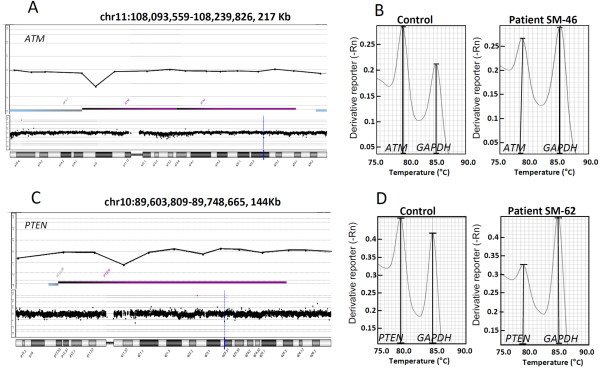
**Array CGH and Duplex qPCR.** Validation of three selected DNA copy number alterations detected using array CGH in HBOC patients. **A**: Chromosome 11 array CGH profile (lower panel) of a cancer patient (SM-46); the vertical blue bar indicates the affected genomic region, which is enlarged in the upper panel showing a deletion in *ATM* exon 4 (upper panel). **B**: Duplex qPCR for the *ATM* exon 4 and a reference gene; the ratio between the patient and control peaks of the melting curve was 0.63, which confirmed a one-copy deletion. **C**: Chromosome 10 array CGH profile (lower panel) of a cancer patient (SM-62) showing a deletion in *PTEN* exon 2 (upper panel); the blue vertical bar in the chromosome indicates the gene region, which is enlarged in the upper panel. **D**: Duplex qPCR for the *PTEN* exon 2 and reference gene; the ratio between the patient and control peaks of the melting curve was 0.64, confirming the one-copy deletion. Coordinates of the enlarged gene region are given according to the UCSC Feb. 2009 (GRCh37-Hg19) assembly.

Among the 120 cancer patients included in this study, 100 had array CGH data available from a previous study [19], which were used for detecting CNVs within the 14 breast cancer susceptibility genes. After a careful visual inspection, two of them were found to harbor CNVs in *PTEN* and *ATM* genes. The exon-4 heterozygous deletion in the *ATM* gene (Patient SM-46, carrier of a pathogenic *BRCA2* mutation, c.K3161*) and exon-2 heterozygous deletion in the *PTEN* gene (Patient SM-62) were confirmed using the duplex qPCR gene dosage method (Figure 4). However, both the array CGH probe and gene dosage PCR primers for the *PTEN* gene were located in the deleted region that has recently been described as a polymorphic chromosomal deletion [21].

## Discussion

In Brazil, data concerning the prevalence of *BRCA1/2* mutations are limited. Previous studies using different selection criteria have reported mutation frequencies ranging from 2.3% to 20% [22-28]. The largest study conducted in Brazil used the protein truncation test (PTT) to evaluate 612 BC cases with high and medium risks of breast cancer and found a mutation carrier prevalence of 3.4% [25]. In the current study, we detected a germ line mutation prevalence of 26% within the *BRCA1/2* genes, *TP53* R337H and *CHEK2* 1100delC in 120 Brazilian patients with clinical criteria for HBOC (16.5% in *BRCA1,* 6% in *BRCA2*, 2.5% in *TP53* and 1% in *CHEK2*). Previous studies have described a mutation detection rate ranging from 8.9 to 43.8% [29-31] in *BRCA1/2*, revealing differences between our cohort and others. Thus, it is important to note that our study was based on an institutional registry and probably does not represent the broad ethnic and socio-economic diversity of the Brazilian population. This can partially explain some inconsistency between different Brazilian studies, in addition to the different screening methods and inclusion criteria for selecting patients.

Regardless of specific populations and ethnic groups, recurrent *BRCA1/2* mutations are rarely detected in hereditary breast cancer. In this sense, a wide range of pathogenic mutations was detected in this series, which is expected for an unrelated cohort of an ethnically mixed population such as that of Brazil. The most frequent mutation identified in this series was the Ashkenazi Jewish 5382insC variant, which was found in approximately 10% of the mutation carriers. This is one of the most common *BRCA1* mutations identified worldwide and is found both among Ashkenazi Jews and women of Slavic origin [32,33].

Splice-site mutations in the *BRCA1* gene are considered to be rare, and thus far, only a few splice-site mutations have been reported in the BIC database. Using transcriptional analysis of the BC tumor harboring the novel mutation c.560 + 2 T > A, we confirmed the presence of an aberrant transcript that was not found in the control sample. Interestingly, this variant was also reported in another series of unrelated young Brazilian patients with a positive family history recently reported by us [28]. Although we were able to show that the mutant allele can produce the aberrant transcript but not able to demonstrate whether the mutant allele was still able to produce the full-length *BRCA1* transcript, this splice site was considered pathogenic because it has been reported that mutations in the highly conserved acceptor or donor sites are pathogenic *per se*[34].

One of the main issues in the molecular diagnosis of *BRCA1/2* mutations is the effect of VUSs in protein function. Several approaches have been used to determine the pathogenicity of VUSs, including the investigation of co-segregation within pedigrees, frequency in healthy controls, lack of co-occurrence with pathogenic mutations, and *in silico* analysis such as amino acid conservation and the severity of amino acid change [35]. According to the criteria adopted in this study, only the p.M784V variant had an uncertain clinical relevance according to the IARC-LOVD database; however, because of the lack of co-segregation in one affected sister and the presence of the variant in a set of 95 healthy individuals (data not shown), this variant is likely to have little or no clinical relevance.

Our results demonstrated that the age at cancer diagnosis had a significant impact on the positive detection rate. In this sense, we found that the group of early-onset breast cancer patients (≤35 yo) is at a higher risk of carrying pathogenic mutations in the *BRCA1/2* genes with a positive detection rate of 35%, which reached 42% in cases with a family history of breast cancer (not shown). In a previous study by our group, patients ≤ 35 years of age showed a mutation rate of 20% in *BRCA1/2* genes with a significant increase of the detection rate in young women with a positive family history (37.5%) [28]. The concordance among these studies in early-onset breast cancer patients strengthens the hypothesis that young Brazilian women with a positive family history are at high risk of being *BRCA*1/2 carriers.

Li-Fraumeni syndrome is inherited in an autosomal dominant manner and is associated with germ line mutations in the *TP53* gene. Despite the broad range of pathogenic mutations in this gene, a specific mutation occurring in the tetramerization domain of the *TP53* gene (p.R337H) has been reported at a high prevalence in southern and southeastern Brazil. Recent studies have identified p.R377H carriers in a variety of tumors, in particular, early breast cancer [36]. In our analysis, the three carriers had breast cancer prior to the age of 50 without a family history of other tumors typical of Li-Fraumeni syndrome. Therefore, due to the high prevalence of the R337H mutation in southeast Brazil, we asserted that a genetic test for this variant is strongly recommended for families matching clinical criteria for HBOC and in whom mutation testing for *BRCA1* and *BRCA2* is negative.

Germ line DNA CNVs have recently been implicated in predisposition to different tumors [17]. In this regard, Rouleau and colleagues [37] used an in-house array CGH platform to search for copy number imbalances in ten genes involved in hereditary breast and ovarian cancer including *BRCA1, BRCA2, CHEK2, BARD1, ATM, RAD50, RAD51, BRIP1, RAP80* and *PALB2.* In a series of 472 patients, they found only three large rearrangements in *BRCA1/2*, two in *CHEK2* and one intronic deletion in *BRIP1*. In our series, with the exception of *RAP80*, all genes were also evaluated for DNA copy number imbalances. We detected four large rearrangements (3% of the cohort), thus confirming the two rearrangements affecting *BRCA1* and revealing two one-exon deletions in the *ATM* and *PTEN* genes. Since we cannot rule out the presence of point mutations in these 14 genes, we can only suggest that germ line CNVs in these genes are at low frequencies. Additionally, CNVs in both *BRCA1* and *BRCA2* genes were also confirmed to be at low frequency in this Brazilian HBOC series.

Germ line point mutations in *ATM* and *PTEN* have been reported to play a role in breast cancer predisposition [38-40]. However, to our knowledge, CNVs within these genes had never been reported in HBOC patients. In the current study, patient SM-46, who was found to carry an exon 4 deletion in the *ATM* gene, also had a pathogenic mutation in the *BRCA2* gene; therefore, the involvement of the *ATM* intragenic deletion with breast cancer predisposition in this particular case remains to be clarified.

Germ line mutations in the *PTEN* gene are associated with the PTEN hamartoma tumor syndrome (PHTS) in which Cowden syndrome (CS) is the most common phenotype. Patients with CS are at an increased risk of a variety of tumors including a 50% increased lifetime risk for breast cancer [41]. Although one of our BC patients presented a *PTEN* exon 2 deletion, recently, Sandell and colleagues described an 899-bp intronic deletion located 58 bp upstream *PTEN* exon 2 (c.80-956_-58del899), which was identified in 4% of British PHTS patients and in 3% of healthy individuals [20]. Apparently, this British alteration is the same found in our BC patient; one of the primer pairs designed for duplex PCR was located within this polymorphic region. Nevertheless, according to Sandell and colleagues, the presence of this polymorphism in healthy individuals, the lack of aberrant splicing and the co-occurrence with known pathogenic mutations indicate that this variant is probably a polymorphism and has no phenotypic effect.

## Conclusions

In summary, this is the most comprehensive *BRCA1/2* mutation screening study of Brazilian BC patients from families with hereditary breast and ovarian cancer. The study demonstrates a high prevalence of *BRCA1* point mutations and low frequency of CNVs within the *BRCA1* and *BRCA2* genes. Moreover, the detection of the *TP53* R337H variant in our series and the fact that this variant has a founder effect in our population prompted us to suggest that all female breast cancer patients with clinical criteria for HBOC and negative for *BRCA1/2* genes should be tested for this variant. Additionally, the identification of CNVs in other breast cancer susceptibility genes revealed the complex genetic basis of this series of 120 unrelated Brazilian women with hereditary breast and ovarian cancer.

## Competing interests

The authors declare that they have no competing interests.

## Authors’ contributions

BMR and DMC conceived the study; FCCS, BCL, MCPF, GTT, ACK and DMC performed and analyzed the experiments. EMMS, MIA and BMR assessed the clinical data and selected patients. BMR and DMC contributed reagents, materials and analysis tools. FCS and DMC wrote and edited the manuscript. BCL, MCPF and GTT edited and revised the manuscript. All authors have read and approved the final version of the manuscript.

## Pre-publication history

The pre-publication history for this paper can be accessed here:

http://www.biomedcentral.com/1471-2350/15/55/prepub

## Supplementary Material

Additional file 1 Complete molecular information of 120 patients. Click here for file

Additional file 2 **MLPA analysis.** A. Electropherograms showing reduced peaks (arrows) of exons 16 and 17 of the *BRCA1* gene in patient SM-03 compared with a control sample (C), characterizing a two-exon deletion. B. Electropherogram obtained from the patient MO-28 showing the amplification (off-scale peak) of exon 24 in the *BRCA1* gene (arrow). Click here for file
