# Supplementary material for: Hereditary breast and ovarian cancer: assessment of point mutations and copy number variations in Brazilian patients
Source: BMC Med Genet. 2014 May 15;15:55. doi: 10.1186/1471-2350-15-55 (PMC4038072; doi:10.1186/1471-2350-15-55)
Supplement: Additional file 1 — Complete molecular information of 120 patients. [file 1471-2350-15-55-S1.doc]

| Article Title: Hereditary Breast and Ovarian Cancer: Assessment of point mutations and copy number variations in Brazilian patients | | | | | | | | | | | |
| --- | --- | --- | --- | --- | --- | --- | --- | --- | --- | --- | --- |
| Journal: BMCMedical Genetics | | | | | | | | | | | |
| Authors: Silva FC, Lisboa BCG, Figueiredo MCP, Torrezan GT, Santos EMM, Krepischi ACV, Rossi BM, Achatz MI , Carraro DM. | | | | | | | | | | | |
| **Corresponding author: Dirce Maria Carraro - Email: dirce.carraro@cipe.accamargo.org.br - Laboratory of Genomics and Molecular Biology, A. C. Camargo Cancer Center, São Paulo, Brazil** | | | | | | | | | | | |
| Supplementary Table S1. Complete molecular information of 120 patients | | | | | | | | | | | |
| ID | Tumor type | Age of diagnosis | Syndrome | Inclusion criteria | Source | Gene | Alteration (BIC database) | Consequence | Exon | Clinically relevant (BIC database) | Clinically relevant (LOVD-IARC) |
| SM-01 | Breast cancer | 39 | HBOC | 2 | Germline | BRCA1 | c.5203delTT | p.Phe1695Cysfs*3 | 18 | Yes | - |
|  |  |  |  |  |  | BRCA2 | c.1342C>A | p.H372N | 10 | No | class 1 |
|  |  |  |  |  |  | BRCA2 | c.[2024T>C] ; [2024T>C] | p.F599S | 10 | unknown | - |
| SM-02 | Breast cancer | 47 | HBOC | 3 | Germline | BRCA1 | c.[2201C>T] ; [2201C>T] | p.S694S | 11 | unknown | - |
|  | Thyroid cancer | 58 |  |  |  | BRCA1 | c.[2430T>C]; [2430T>C] | p.L771L | 11 | unknown | - |
|  |  |  |  |  |  | BRCA1 | c.[2731C>T] ; [2731C>T] | p.P871L | 11 | No | - |
|  |  |  |  |  |  | BRCA1 | c.[3232A>G] ; [3232A>G] | p.E1038G | 11 | No | class 1 |
|  |  |  |  |  |  | BRCA1 | c.[3667A>G] ; [3667A>G] | p.K1183R | 11 | No | class 1 |
|  |  |  |  |  |  | BRCA1 | c.[4427T>C]; [4427T>C] | p.S1436S | 13 | unknown | - |
|  |  |  |  |  |  | BRCA1 | c.[4956A>G] ; [4956A>G] | p.S1613G | 16 | No | class 1 |
|  |  |  |  |  |  | BRCA2 | c.1093A>C | p.N289H | 10 | No | - |
|  |  |  |  |  |  | BRCA2 | c.[1342C>A] ; [1342C>A] | p.H372N | 10 | No | class 1 |
|  |  |  |  |  |  | BRCA2 | c.1593A>G | p.S455S | 10 | No | - |
|  |  |  |  |  |  | BRCA2 | c.[2024T>C] ; [2024T>C] | p.F599S | 10 | unknown | - |
|  |  |  |  |  |  | BRCA2 | c.2457T>C | p.H743H | 11 | unknown | - |
|  |  |  |  |  |  | BRCA2 | c.3199A>G | p.N991D | 11 | No | - |
|  |  |  |  |  |  | BRCA2 | c.4035T>C | p.V1269V | 11 | No | - |
| SM-03 | Breast cancer | 36 | HBOC | 6 | Germline | BRCA1 | c.2201C>T | p.S694S | 11 | unknown | - |
|  | Ovarian cancer | 36 |  |  |  | BRCA1 | c.2430T>C | p.L771L | 11 | unknown | - |
|  |  |  |  |  |  | BRCA1 | c.2731C>T | p.P871L | 11 | No | - |
|  |  |  |  |  |  | BRCA1 | c.3232A>G | p.E1038G | 11 | No | class 1 |
|  |  |  |  |  |  | BRCA1 | c.3667A>G | p.K1183R | 11 | No | class 1 |
|  |  |  |  |  |  | BRCA1 | c.4427T>C | p.S1436S | 13 | unknown | - |
|  |  |  |  |  |  | BRCA1 | Exon 16-17 deletion | - |  | Yes | - |
|  |  |  |  |  |  | BRCA2 | c.203G>A | - | 5' UTR | No | - |
|  |  |  |  |  |  | BRCA2 | c.[1342C>A] ; [1342C>A] | p.H372N | 10 | No | class 1 |
|  |  |  |  |  |  | BRCA2 | c.[2024T>C] ; [2024T>C] | p.F599S | 10 | unknown | - |
|  |  |  |  |  |  | BRCA2 | c.3624A>G | p.K1132K | 11 | No | - |
|  |  |  |  |  |  | BRCA2 | c.4035T>C | p.V1269V | 11 | No | - |
|  |  |  |  |  |  | BRCA2 | c.5972C>T | p.T1915M | 11 | Unknown | - |
|  |  |  |  |  |  | BRCA2 | c.7470A>G | p.S2414S | 14 | No | - |
| SM-04 | Breast cancer | 48 | HBOC | 3 | Germline | BRCA1 | c.1186A>G | p.Q356R | 11 | Unknown | class 1 |
|  |  |  |  |  |  | BRCA2 | c.[1342C>A] ; [1342C>A] | p.H372N | 10 | No | class 1 |
|  |  |  |  |  |  | BRCA2 | c.[2024T>C] ; [2024T>C] | p.F599S | 10 | unknown | - |
| SM-05 | Breast cancer | 36 | HBOC | 2 | Germline | BRCA1 | c.[2731C>T] ; [2731C>T] | p.P871L | 11 | No | - |
|  |  |  |  |  |  | BRCA1 | c.3537A>G | p.S1140G | 11 | No | class 1 |
|  |  |  |  |  |  | BRCA2 | c.[2024T>C] ; [2024T>C] | p.F599S | 10 | unknown | - |
|  |  |  |  |  |  | BRCA2 | c.9058A>T | p.I2944F | 22 | Unknown | - |
| SM-07 | Breast cancer | 54 | HBOC | 4 | Germline | BRCA2 | c.203G>A | - | 5' UTR | No | - |
|  | Colorectal cancer | 56 |  |  |  | BRCA2 | c.1342C>A | p.H372N | 10 | No | class 1 |
|  | GIST | 61 |  |  |  | BRCA2 | c.1093A>C | p.N289H | 10 | No | - |
|  |  |  |  |  |  | BRCA2 | c.1593A>G | p.S455S | 10 | No | - |
|  |  |  |  |  |  | BRCA2 | c.[2024T>C] ; [2024T>C] | p.F599S | 10 | unknown | - |
|  |  |  |  |  |  | BRCA2 | c.2457T>C | p.H743H | 11 | unknown | - |
|  |  |  |  |  |  | BRCA2 | c.3199A>G | p.N991D | 11 | No | - |
|  |  |  |  |  |  | BRCA2 | c.3624A>G | p.K1132K | 11 | No | - |
|  |  |  |  |  |  | BRCA2 | c.7470A>G | p.S2414S | 14 | No | - |
| SM-08 | Breast cancer | 33 | HBOC | 7 | Germline | BRCA2 | c.203G>A | - | 5' UTR | No | - |
|  |  |  |  |  |  | BRCA2 | c.1342C>A | p.H372N | 10 | No | class 1 |
|  |  |  |  |  |  | BRCA2 | c.3624A>G | p.K1132K | 11 | No | - |
|  |  |  |  |  |  | BRCA2 | c.6174delT | p.Ser1982Argfs*22 | 11 | Yes | - |
| SM-09 | Breast cancer | 44 | HBOC | 1 | Germline | BRCA2 | c.1093A>C | p.N289H | 10 | No | - |
|  | Colorectal cancer | 48 |  |  |  | BRCA2 | c.1342C>A | p.H372N | 10 | No | class 1 |
|  |  |  |  |  |  | BRCA2 | c.[2024T>C] ; [2024T>C] | p.F599S | 10 | unknown | - |
|  |  |  |  |  |  | BRCA2 | c.3624A>G | p.K1132K | 11 | No | - |
|  |  |  |  |  |  | BRCA2 | c.4035T>C | p.V1269V | 11 | No | - |
|  |  |  |  |  |  | BRCA2 | c.7470A>G | p.S2414S | 14 | No | - |
| SM-10 | Breast cancer | 52 | HBOC | 4 | Germline | BRCA1 | c.5002T>C | p.M1628T | 16 | No | class 1 |
|  |  |  |  |  |  | BRCA2 | c.203G>A | - | 5' UTR | No | - |
|  |  |  |  |  |  | BRCA2 | c.1342C>A | p.H372N | 10 | No | class 1 |
|  |  |  |  |  |  | BRCA2 | c.[2024T>C] ; [2024T>C] | p.F599S | 10 | unknown | - |
|  |  |  |  |  |  | BRCA2 | c.3624A>G | p.K1132K | 11 | No | - |
|  |  |  |  |  |  | BRCA2 | c.4035T>C | p.V1269V | 11 | No | - |
|  |  |  |  |  |  | BRCA2 | c.7470A>G | p.S2414S | 14 | No | - |
| SM-11 | Ovarian cancer | 40 | HBOC | 6 | Germline | BRCA1 | c.2731C>T | p.P871L | 11 | No | - |
|  | Breast cancer | 53 |  |  |  | BRCA2 | c.203G>A | - | 5' UTR | No | - |
|  |  |  |  |  |  | BRCA2 | c.1342C>A | p.H372N | 10 | No | class 1 |
|  |  |  |  |  |  | BRCA2 | c.[2024T>C] ; [2024T>C] | p.F599S | 10 | unknown | - |
|  |  |  |  |  |  | BRCA2 | c.3624A>G | p.K1132K | 11 | No | - |
|  |  |  |  |  |  | BRCA2 | c.4035T>C | p.V1269V | 11 | No | - |
|  |  |  |  |  |  | BRCA2 | c.7470A>G | p.S2414S | 14 | No | - |
| SM-12 | Colorectal cancer | 41 | HBOC | 6 | Germline | BRCA1 | c.[203G>A] ; [203G>A] | - | 5' UTR | No | - |
|  | Breast cancer | 42 |  |  |  | BRCA2 | c.[1342C>A] ; [1342C>A] | p.H372N | 10 | No | class 1 |
|  | Ovarian cancer | 43 |  |  |  | BRCA2 | c.[2024T>C] ; [2024T>C] | p.F599S | 10 | unknown | - |
|  |  |  |  |  |  | BRCA2 | c.[3624A>G]; [3624A>G] | p.K1132K | 11 | No | - |
|  |  |  |  |  |  | BRCA2 | c.[7470A>G] ; [7470A>G] | p.S2414S | 14 | No | - |
| SM-13 | Breast cancer | 44 | HBOC | 2 | Germline | BRCA1 | c.2201C>T | p.S694S | 11 | unknown | - |
|  |  |  |  |  |  | BRCA1 | c.2430T>C | p.L771L | 11 | unknown | - |
|  |  |  |  |  |  | BRCA1 | c.2731C>T | p.P871L | 11 | No | - |
|  |  |  |  |  |  | BRCA1 | c.3232A>G | p.E1038G | 11 | No | class 1 |
|  |  |  |  |  |  | BRCA1 | c.3667A>G | p.K1183R | 11 | No | class 1 |
|  |  |  |  |  |  | BRCA1 | c.4427T>C | p.S1436S | 13 | unknown | - |
|  |  |  |  |  |  | BRCA1 | c.4956A>G | p.S1613G | 16 | No | class 1 |
|  |  |  |  |  |  | BRCA2 | c.[2024T>C] ; [2024T>C] | p.F599S | 10 | unknown | - |
| SM-14 | Breast cancer | 45 | HBOC | 1 | Germline | BRCA1 | c.1186A>G | p.Q356R | 11 | Unknown | class 1 |
|  | Colorectal cancer | 50 |  |  |  | BRCA1 | c.4654G>T | p.S1512I | 15 | No | class 1 |
|  |  |  |  |  |  | BRCA2 | c.203G>A | - | 5' UTR | No | - |
|  |  |  |  |  |  | BRCA2 | c.1093A>C | p.N289H | 10 | No | - |
|  |  |  |  |  |  | BRCA2 | c.1342C>A | p.H372N | 10 | No | class 1 |
|  |  |  |  |  |  | BRCA2 | c.1593A>G | p.S455S | 10 | No | - |
|  |  |  |  |  |  | BRCA2 | c.[2024T>C] ; [2024T>C] | p.F599S | 10 | unknown | - |
|  |  |  |  |  |  | BRCA2 | c.2457T>C | p.H743H | 11 | unknown | - |
|  |  |  |  |  |  | BRCA2 | c.3199A>G | p.N991D | 11 | No | - |
|  |  |  |  |  |  | BRCA2 | c.3624A>G | p.K1132K | 11 | No | - |
|  |  |  |  |  |  | BRCA2 | c.7470A>G | p.S2414S | 14 | No | - |
| SM-15 | Breast cancer | 48 | HBOC | 3 | Germline | BRCA1 | c.2201C>T | p.S694S | 11 | unknown | - |
|  |  |  |  |  |  | BRCA1 | c.2430T>C | p.L771L | 11 | unknown | - |
|  |  |  |  |  |  | BRCA1 | c.2731C>T | p.P871L | 11 | No | - |
|  |  |  |  |  |  | BRCA1 | c.3232A>G | p.E1038G | 11 | No | class 1 |
|  |  |  |  |  |  | BRCA1 | c.3667A>G | p.K1183R | 11 | No | class 1 |
|  |  |  |  |  |  | BRCA1 | c.4427T>C | p.S1436S | 13 | unknown | - |
|  |  |  |  |  |  | BRCA1 | c.4956A>G | p.S1613G | 16 | No | class 1 |
|  |  |  |  |  |  | BRCA2 | c.203G>A | - | 5' UTR | No | - |
|  |  |  |  |  |  | BRCA2 | c.1342C>A | p.H372N | 10 | No | class 1 |
|  |  |  |  |  |  | BRCA2 | c.[2024T>C] ; [2024T>C] | p.F599S | 10 | unknown | - |
|  |  |  |  |  |  | BRCA2 | c.3624A>G | p.K1132K | 11 | No | - |
| SM-16 | Breast cancer | 33 | HBOC | 2 | Germline | BRCA1 | c.2201C>T | p.S694S | 11 | unknown | - |
|  |  |  |  |  |  | BRCA1 | c.2430T>C | p.L771L | 11 | unknown | - |
|  |  |  |  |  |  | BRCA1 | c.2731C>T | p.P871L | 11 | No | - |
|  |  |  |  |  |  | BRCA1 | c.3232A>G | p.E1038G | 11 | No | class 1 |
|  |  |  |  |  |  | BRCA1 | c.3667A>G | p.K1183R | 11 | No | class 1 |
|  |  |  |  |  |  | BRCA1 | c.4427T>C | p.S1436S | 13 | unknown | - |
|  |  |  |  |  |  | BRCA1 | c.4956A>G | p.S1613G | 16 | No | class 1 |
|  |  |  |  |  |  | BRCA2 | c.203G>A | - | 5' UTR | No | - |
|  |  |  |  |  |  | BRCA2 | c.1342C>A | p.H372N | 10 | No | class 1 |
|  |  |  |  |  |  | BRCA2 | c.[2024T>C] ; [2024T>C] | p.F599S | 10 | unknown | - |
|  |  |  |  |  |  | BRCA2 | c.3624A>G | p.K1132K | 11 | No | - |
|  |  |  |  |  |  | BRCA2 | c.5427C>T | p.S1733S | 11 | No | - |
|  |  |  |  |  |  | BRCA2 | c.7470A>G | p.S2414S | 14 | No | - |
| SM-17 | Ovarian cancer | 49 | HBOC | 6 | Germline | BRCA1 | c.3376T>G | p.L1086* | 11 | Yes | - |
|  | Breast cancer | 51 |  |  |  | BRCA2 | c.1342C>A | p.H372N | 10 | No | class 1 |
|  |  |  |  |  |  | BRCA2 | c.[2024T>C] ; [2024T>C] | p.F599S | 10 | unknown | - |
| SM-18 | Breast cancer | 61 | HBOC | 4 | Germline | BRCA1 | c.2201C>T | p.S694S | 11 | unknown | - |
|  |  |  |  |  |  | BRCA1 | c.2430T>C | p.L771L | 11 | unknown | - |
|  |  |  |  |  |  | BRCA1 | c.2731C>T | p.P871L | 11 | No | - |
|  |  |  |  |  |  | BRCA1 | c.3232A>G | p.E1038G | 11 | No | class 1 |
|  |  |  |  |  |  | BRCA1 | c.3667A>G | p.K1183R | 11 | No | class 1 |
|  |  |  |  |  |  | BRCA1 | c.4427T>C | p.S1436S | 13 | unknown | - |
|  |  |  |  |  |  | BRCA1 | c.4956A>G | p.S1613G | 16 | No | class 1 |
|  |  |  |  |  |  | BRCA2 | c.[203G>A] ; [203G>A] | - | 5' UTR | No | - |
|  |  |  |  |  |  | BRCA2 | c.[1342C>A] ; [1342C>A] | p.H372N | 10 | No | class 1 |
|  |  |  |  |  |  | BRCA2 | c.[2024T>C] ; [2024T>C] | p.F599S | 10 | unknown | - |
|  |  |  |  |  |  | BRCA2 | c.[3624A>G]; [3624A>G] | p.K1132K | 11 | No | - |
|  |  |  |  |  |  | BRCA2 | c.[7470A>G] ; [7470A>G] | p.S2414S | 14 | No | - |
|  |  |  |  |  |  | BRCA2 | c.8034-14T/C | - | i16 | No | - |
| SM-19 | Breast cancer | 60 | HBOC | 4 |  | BRCA1 | c.3238G>A | p.S1040N | 11 | No | class 1 |
|  | Colorectal cancer | 60 |  |  |  | BRCA2 | c.1093A>C | p.N289H | 10 | No | - |
|  |  |  |  |  |  | BRCA2 | c.1342C>A | p.H372N | 10 | No | class 1 |
|  |  |  |  |  |  | BRCA2 | c.1593A>G | p.S455S | 10 | No | - |
|  |  |  |  |  |  | BRCA2 | c.[2024T>C] ; [2024T>C] | p.F599S | 10 | unknown | - |
|  |  |  |  |  |  | BRCA2 | c.2457T>C | p.H743H | 11 | unknown | - |
|  |  |  |  |  |  | BRCA2 | c.3199A>G | p.N991D | 11 | No | - |
|  |  |  |  |  |  | BRCA2 | c.9079G>A | p.A2951T | 22 | No | - |
| SM-20 | Ovarian cancer | 35 | HBOC | 8 | Germline | BRCA1 | c.5002T>C | p.M1628T | 16 | No | class 1 |
|  |  |  |  |  |  | BRCA2 | c.203G>A | - | 5' UTR | No | - |
|  |  |  |  |  |  | BRCA2 | c.1342C>A | p.H372N | 10 | No | class 1 |
|  |  |  |  |  |  | BRCA2 | c.[2024T>C] ; [2024T>C] | p.F599S | 10 | unknown | - |
|  |  |  |  |  |  | BRCA2 | c.2578A>G | p.M784V | 11 | Unknown | class 3 |
|  |  |  |  |  |  | BRCA2 | c.3624A>G | p.K1132K | 11 | No | - |
|  |  |  |  |  |  | BRCA2 | c.7470A>G | p.S2414S | 14 | No | - |
| SM-21 | Ovarian cancer | 29 | HBOC | 8 | Germline | BRCA1 | c.1186A>G | p.Q356R | 11 | Unknown | class 1 |
|  |  |  |  |  |  | BRCA1 | c.2201C>T | p.S694S | 11 | unknown | - |
|  |  |  |  |  |  | BRCA1 | c.2430T>C | p.L771L | 11 | unknown | - |
|  |  |  |  |  |  | BRCA1 | c.2731C>T | p.P871L | 11 | No | - |
|  |  |  |  |  |  | BRCA1 | c.3232A>G | p.E1038G | 11 | No | class 1 |
|  |  |  |  |  |  | BRCA1 | c.3667A>G | p.K1183R | 11 | No | class 1 |
|  |  |  |  |  |  | BRCA1 | c.4427T>C | p.S1436S | 13 | unknown | - |
|  |  |  |  |  |  | BRCA1 | c.4956A>G | p.S1613G | 16 | No | class 1 |
|  |  |  |  |  |  | BRCA2 | c.1342C>A | p.H372N | 10 | No | class 1 |
|  |  |  |  |  |  | BRCA2 | c.[2024T>C] ; [2024T>C] | p.F599S | 10 | unknown | - |
| SM-22 | Breast cancer | 62 | HBOC | 4 | Germline | BRCA1 | c.2201C>T | p.S694S | 11 | unknown | - |
|  |  |  |  |  |  | BRCA1 | c.2430T>C | p.L771L | 11 | unknown | - |
|  |  |  |  |  |  | BRCA1 | c.2731C>T | p.P871L | 11 | No | - |
|  |  |  |  |  |  | BRCA1 | c.3232A>G | p.E1038G | 11 | No | class 1 |
|  |  |  |  |  |  | BRCA1 | c.3667A>G | p.K1183R | 11 | No | class 1 |
|  |  |  |  |  |  | BRCA1 | c.4427T>C | p.S1436S | 13 | unknown | - |
|  |  |  |  |  |  | BRCA1 | c.4956A>G | p.S1613G | 16 | No | class 1 |
|  |  |  |  |  |  | BRCA2 | c.[203G>A] ; [203G>A] | - | 5' UTR | No | - |
|  |  |  |  |  |  | BRCA2 | c.[1342C>A] ; [1342C>A] | p.H372N | 10 | No | class 1 |
|  |  |  |  |  |  | BRCA2 | c.[2024T>C] ; [2024T>C] | p.F599S | 10 | unknown | - |
|  |  |  |  |  |  | BRCA2 | c.[3624A>G]; [3624A>G] | p.K1132K | 11 | No | - |
|  |  |  |  |  |  | BRCA2 | c.[7470A>G] ; [7470A>G] | p.S2414S | 14 | No | - |
| SM-23 | Colorectal cancer | 61 | HBOC | 4 | Germline | BRCA1 | c.2201C>T | p.S694S | 11 | unknown | - |
|  | Breast cancer | 63 |  |  |  | BRCA1 | c.2430T>C | p.L771L | 11 | unknown | - |
|  |  |  |  |  |  | BRCA1 | c.2731C>T | p.P871L | 11 | No | - |
|  |  |  |  |  |  | BRCA1 | c.3232A>G | p.E1038G | 11 | No | class 1 |
|  |  |  |  |  |  | BRCA1 | c.3667A>G | p.K1183R | 11 | No | class 1 |
|  |  |  |  |  |  | BRCA1 | c.4427T>C | p.S1436S | 13 | unknown | - |
|  |  |  |  |  |  | BRCA1 | c.4956A>G | p.S1613G | 16 | No | class 1 |
|  |  |  |  |  |  | BRCA2 | c.1342C>A | p.H372N | 10 | No | class 1 |
|  |  |  |  |  |  | BRCA2 | c.[2024T>C] ; [2024T>C] | p.F599S | 10 | unknown | - |
| SM-24 | Breast cancer | 42 | HBOC | 2 | Germline | BRCA1 | c.233G>A | p.K38K | 3 | No | - |
|  |  |  |  |  |  | BRCA1 | c.2201C>T | p.S694S | 11 | unknown | - |
|  |  |  |  |  |  | BRCA1 | c.2430T>C | p.L771L | 11 | unknown | - |
|  |  |  |  |  |  | BRCA1 | c.2731C>T | p.P871L | 11 | No | - |
|  |  |  |  |  |  | BRCA1 | c.3232A>G | p.E1038G | 11 | No | class 1 |
|  |  |  |  |  |  | BRCA1 | c.3667A>G | p.K1183R | 11 | No | class 1 |
|  |  |  |  |  |  | BRCA1 | c.4427T>C | p.S1436S | 13 | unknown | - |
|  |  |  |  |  |  | BRCA1 | c.4956A>G | p.S1613G | 16 | No | class 1 |
|  |  |  |  |  |  | BRCA2 | c.203G>A | - | 5' UTR | No | - |
|  |  |  |  |  |  | BRCA2 | c.1093A>C | p.N289H | 10 | No | - |
|  |  |  |  |  |  | BRCA2 | c.1192A>C | p.K322Q | 10 | Unknown | - |
|  |  |  |  |  |  | BRCA2 | c.[1342C>A] ; [1342C>A] | p.H372N | 10 | No | class 1 |
|  |  |  |  |  |  | BRCA2 | c.1593A>G | p.S455S | 10 | No | - |
|  |  |  |  |  |  | BRCA2 | c.[2024T>C] ; [2024T>C] | p.F599S | 10 | unknown | - |
|  |  |  |  |  |  | BRCA2 | c.2457T>C | p.H743H | 11 | unknown | - |
|  |  |  |  |  |  | BRCA2 | c.2578A>G | p.M784V | 11 | Unknown | class 3 |
|  |  |  |  |  |  | BRCA2 | c.3199A>G | p.N991D | 11 | No | - |
|  |  |  |  |  |  | BRCA2 | c.3624A>G | p.K1132K | 11 | No | - |
|  |  |  |  |  |  | BRCA2 | c.7470A>G | p.S2414S | 14 | No | - |
| SM-25 | Breast cancer | 69 | HBOC | 4 | Germline | BRCA1 | c.120A>G | p.M1V | 2 | Yes | - |
|  |  |  |  |  |  | BRCA1 | c.2196G>A | p.D693N | 11 | No | class 1 |
|  |  |  |  |  |  | BRCA1 | c.2201C>T | p.S694S | 11 | unknown | - |
|  |  |  |  |  |  | BRCA1 | c.2430T>C | p.L771L | 11 | unknown | - |
|  |  |  |  |  |  | BRCA1 | c.2731C>T | p.P871L | 11 | No | - |
|  |  |  |  |  |  | BRCA1 | c.3232A>G | p.E1038G | 11 | No | class 1 |
|  |  |  |  |  |  | BRCA1 | c.3667A>G | p.K1183R | 11 | No | class 1 |
|  |  |  |  |  |  | BRCA1 | c.4427T>C | p.S1436S | 13 | unknown | - |
|  |  |  |  |  |  | BRCA1 | c.4956A>G | p.S1613G | 16 | No | class 1 |
|  |  |  |  |  |  | BRCA2 | c.[203G>A] ; [203G>A] | - | 5' UTR | No | - |
|  |  |  |  |  |  | BRCA2 | c.[1342C>A] ; [1342C>A] | p.H372N | 10 | No | class 1 |
|  |  |  |  |  |  | BRCA2 | c.[2024T>C] ; [2024T>C] | p.F599S | 10 | unknown | - |
|  |  |  |  |  |  | BRCA2 | c.[3624A>G]; [3624A>G] | p.K1132K | 11 | No | - |
|  |  |  |  |  |  | BRCA2 | c.[7470A>G] ; [7470A>G] | p.S2414S | 14 | No | - |
| SM-28 | Breast cancer | 34 | HBOC | 1 | Germline | BRCA2 | c.1342C>A | p.H372N | 10 | No | class 1 |
|  | Bladder cancer | 51 |  |  |  | BRCA2 | c.[2024T>C] ; [2024T>C] | p.F599S | 10 | unknown | - |
| SM-29 | Breast cancer | 45 | HBOC | 2 | Germline | BRCA1 | c.2201C>T | p.S694S | 11 | unknown | - |
|  |  | ou40 |  |  |  | BRCA1 | c.2430T>C | p.L771L | 11 | unknown | - |
|  |  |  |  |  |  | BRCA1 | c.2731C>T | p.P871L | 11 | No | - |
|  |  |  |  |  |  | BRCA1 | c.3232A>G | p.E1038G | 11 | No | class 1 |
|  |  |  |  |  |  | BRCA1 | c.3667A>G | p.K1183R | 11 | No | class 1 |
|  |  |  |  |  |  | BRCA1 | c.4427T>C | p.S1436S | 13 | unknown | - |
|  |  |  |  |  |  | BRCA1 | c.4956A>G | p.S1613G | 16 | No | class 1 |
|  |  |  |  |  |  | BRCA2 | c.[2024T>C] ; [2024T>C] | p.F599S | 10 | unknown | - |
| SM-30 | Breast cancer | 56 | HBOC | 4 | Germline | BRCA1 | c.1186A>G | p.Q356R | 11 | Unknown | class 1 |
|  | Colorectal cancer | 62 |  |  |  | BRCA1 | c.3830A>G | p.I1237M | 11 | Unknown | - |
|  |  |  |  |  |  | BRCA1 | c.4962G>A | p.A1615T | 16 | Unknown | - |
|  |  |  |  |  |  | BRCA2 | c.203G>A | - | 5' UTR | No | - |
|  |  |  |  |  |  | BRCA2 | c.353A>G | p.Y42C | 3 | No | class 1 |
|  |  |  |  |  |  | BRCA2 | c.[1342C>A] ; [1342C>A] | p.H372N | 10 | No | class 1 |
|  |  |  |  |  |  | BRCA2 | c.[2024T>C] ; [2024T>C] | p.F599S | 10 | unknown | - |
|  |  |  |  |  |  | BRCA2 | c.3624A>G | p.K1132K | 11 | No | - |
|  |  |  |  |  |  | BRCA2 | c.4035T>C | p.V1269V | 11 | No | - |
|  |  |  |  |  |  | BRCA2 | c.7470A>G | p.S2414S | 14 | No | - |
| SM-31 | Breast cancer | 49 | HBOC | 3 | Germline | BRCA1 | c.1186A>G | p.Q356R | 11 | Unknown | class 1 |
|  |  |  |  |  |  | BRCA2 | c.203G>A | - | 5' UTR | No | - |
|  |  |  |  |  |  | BRCA2 | c.[1342C>A] ; [1342C>A] | p.H372N | 10 | No | class 1 |
|  |  |  |  |  |  | BRCA2 | c.[2024T>C] ; [2024T>C] | p.F599S | 10 | unknown | - |
|  |  |  |  |  |  | BRCA2 | c.3624A>G | p.K1132K | 11 | No | - |
|  |  |  |  |  |  | BRCA2 | c.8377G>T | p.A2717S | 18 | No | class 1 |
|  |  |  |  |  |  | TP53 | c.1010G>A | p.R337H | 10 | Yes | - |
| SM-32 | Breast cancer | 37 | HBOC | 2 | Germline | BRCA1 | c.[2201C>T] ; [2201C>T] | p.S694S | 11 | unknown | - |
|  |  |  |  |  |  | BRCA1 | c.[2430T>C]; [2430T>C] | p.L771L | 11 | unknown | - |
|  |  |  |  |  |  | BRCA1 | c.[2731C>T] ; [2731C>T] | p.P871L | 11 | No | - |
|  |  |  |  |  |  | BRCA1 | c.[3232A>G] ; [3232A>G] | p.E1038G | 11 | No | class 1 |
|  |  |  |  |  |  | BRCA1 | c.[3667A>G] ; [3667A>G] | p.K1183R | 11 | No | class 1 |
|  |  |  |  |  |  | BRCA1 | c.[4427T>C]; [4427T>C] | p.S1436S | 13 | unknown | - |
|  |  |  |  |  |  | BRCA1 | c.[4956A>G] ; [4956A>G] | p.S1613G | 16 | No | class 1 |
|  |  |  |  |  |  | BRCA2 | c.1093A>C | p.N289H | 10 | No | - |
|  |  |  |  |  |  | BRCA2 | c.[1342C>A] ; [1342C>A] | p.H372N | 10 | No | class 1 |
|  |  |  |  |  |  | BRCA2 | c.[2024T>C] ; [2024T>C] | p.F599S | 10 | unknown | - |
|  |  |  |  |  |  | BRCA2 | c.2457T>C | p.H743H | 11 | unknown | - |
|  |  |  |  |  |  | BRCA2 | c.3199A>G | p.N991D | 11 | No | - |
| SM-34 | Breast cancer | 56 | HBOC | 4 | Germline | BRCA1 | c.2196G>A | p.D693N | 11 | No | class 1 |
|  | Colorectal cancer | 58 |  |  |  | BRCA1 | c.[2201C>T] ; [2201C>T] | p.S694S | 11 | unknown | - |
|  |  |  |  |  |  | BRCA1 | c.[2430T>C]; [2430T>C] | p.L771L | 11 | unknown | - |
|  |  |  |  |  |  | BRCA1 | c.[2731C>T] ; [2731C>T] | p.P871L | 11 | No | - |
|  |  |  |  |  |  | BRCA1 | c.[3232A>G] ; [3232A>G] | p.E1038G | 11 | No | class 1 |
|  |  |  |  |  |  | BRCA1 | c.[3667A>G] ; [3667A>G] | p.K1183R | 11 | No | class 1 |
|  |  |  |  |  |  | BRCA1 | c.[4427T>C]; [4427T>C] | p.S1436S | 13 | unknown | - |
|  |  |  |  |  |  | BRCA1 | c.[4956A>G] ; [4956A>G] | p.S1613G | 16 | No | class 1 |
|  |  |  |  |  |  | BRCA1 | c.5075G>A | p.M1652I | 16 | No | class 1 |
|  |  |  |  |  |  | BRCA2 | c.[2024T>C] ; [2024T>C] | p.F599S | 10 | unknown | - |
| SM-35 | Breast cancer | 44 | HBOC | 2 | Germline | BRCA1 | c.2201C>T | p.S694S | 11 | unknown | - |
|  |  |  |  |  |  | BRCA1 | c.2430T>C | p.L771L | 11 | unknown | - |
|  |  |  |  |  |  | BRCA1 | c.2731C>T | p.P871L | 11 | No | - |
|  |  |  |  |  |  | BRCA1 | c.3232A>G | p.E1038G | 11 | No | class 1 |
|  |  |  |  |  |  | BRCA1 | c.3667A>G | p.K1183R | 11 | No | class 1 |
|  |  |  |  |  |  | BRCA1 | c.4427T>C | p.S1436S | 13 | unknown | - |
|  |  |  |  |  |  | BRCA1 | c.4956A>G | p.S1613G | 16 | No | class 1 |
|  |  |  |  |  |  | BRCA2 | c.1342C>A | p.H372N | 10 | No | class 1 |
|  |  |  |  |  |  | BRCA2 | c.2016T>C | p.D596D | 10 | No | - |
|  |  |  |  |  |  | BRCA2 | c.[2024T>C] ; [2024T>C] | p.F599S | 10 | unknown | - |
|  |  |  |  |  |  | BRCA2 | c.3624A>G | p.K1132K | 11 | No | - |
|  |  |  |  |  |  | BRCA2 | c.7470A>G | p.S2414S | 14 | No | - |
| SM-36 | Breast cancer | 49 | HBOC | 3 | Germline | BRCA1 | c.3238G>A | p.S1040N | 11 | No | class 1 |
|  |  |  |  |  |  | BRCA2 | c.1342C>A | p.H372N | 10 | No | class 1 |
|  |  |  |  |  |  | BRCA2 | c.[2024T>C] ; [2024T>C] | p.F599S | 10 | unknown | - |
|  |  |  |  |  |  | TP53 | c.1010G>A | p.R337H | 10 | Yes | - |
| SM-37 | Colorectal cancer | 52 | HBOC | 4 |  | BRCA1 | c.2201C>T | p.S694S | 11 | unknown | - |
|  | Breast cancer | 73 |  |  |  | BRCA1 | c.2430T>C | p.L771L | 11 | unknown | - |
|  |  |  |  |  |  | BRCA1 | c.2731C>T | p.P871L | 11 | No | - |
|  |  |  |  |  |  | BRCA1 | c.3232A>G | p.E1038G | 11 | No | class 1 |
|  |  |  |  |  |  | BRCA1 | c.3667A>G | p.K1183R | 11 | No | class 1 |
|  |  |  |  |  |  | BRCA1 | c.4427T>C | p.S1436S | 13 | unknown | - |
|  |  |  |  |  |  | BRCA1 | c.4956A>G | p.S1613G | 16 | No | class 1 |
|  |  |  |  |  |  | BRCA1 | c.5467T>C | p.M1783T | 22 | Unknown | - |
|  |  |  |  |  |  | BRCA2 | c.[1342C>A] ; [1342C>A] | p.H372N | 10 | No | class 1 |
|  |  |  |  |  |  | BRCA2 | c.[2024T>C] ; [2024T>C] | p.F599S | 10 | unknown | - |
|  |  |  |  |  |  | BRCA2 | c.3624A>G | p.K1132K | 11 | No | - |
|  |  |  |  |  |  | BRCA2 | c.6575A>G | p.H2116R | 11 | No | - |
|  |  |  |  |  |  | BRCA2 | c.7470A>G | p.S2414S | 14 | No | - |
| SM-38 | Breast cancer | 42 | HBOC | 5 | Germline | BRCA1 | c.2196G>A | p.D693N | 11 | No | class 1 |
|  | Breast cancer | 47 |  |  |  | BRCA1 | c.2201C>T | p.S694S | 11 | unknown | - |
|  |  |  |  |  |  | BRCA1 | c.2430T>C | p.L771L | 11 | unknown | - |
|  |  |  |  |  |  | BRCA1 | c.2731C>T | p.P871L | 11 | No | - |
|  |  |  |  |  |  | BRCA1 | c.3232A>G | p.E1038G | 11 | No | class 1 |
|  |  |  |  |  |  | BRCA1 | c.3667A>G | p.K1183R | 11 | No | class 1 |
|  |  |  |  |  |  | BRCA1 | c.4427T>C | p.S1436S | 13 | unknown | - |
|  |  |  |  |  |  | BRCA1 | c.4956A>G | p.S1613G | 16 | No | class 1 |
|  |  |  |  |  |  | BRCA2 | c.1342C>A | p.H372N | 10 | No | class 1 |
|  |  |  |  |  |  | BRCA2 | c.[2024T>C] ; [2024T>C] | p.F599S | 10 | unknown | - |
|  |  |  |  |  |  | BRCA2 | c.4035T>C | p.V1269V | 11 | No | - |
| SM-39 | Breast cancer | 45 | HBOC | 2 | Germline | BRCA1 | c.233G>A | p.K38K | 3 | No | - |
|  |  |  |  |  |  | BRCA2 | c.[203G>A] ; [203G>A] | - | 5' UTR | No | - |
|  |  |  |  |  |  | BRCA2 | c.[1342C>A] ; [1342C>A] | p.H372N | 10 | No | class 1 |
|  |  |  |  |  |  | BRCA2 | c.[2024T>C] ; [2024T>C] | p.F599S | 10 | unknown | - |
|  |  |  |  |  |  | BRCA2 | c.2578A>G | p.M784V | 11 | Unknown | class 3 |
|  |  |  |  |  |  | BRCA2 | c.[3624A>G]; [3624A>G] | p.K1132K | 11 | No | - |
|  |  |  |  |  |  | BRCA2 | c.[7470A>G] ; [7470A>G] | p.S2414S | 14 | No | - |
| SM-40 | Breast cancer | 51 | HBOC | 4 | Germline | BRCA1 | c.2201C>T | p.S694S | 11 | unknown | - |
|  |  |  |  |  |  | BRCA1 | c.2430T>C | p.L771L | 11 | unknown | - |
|  |  |  |  |  |  | BRCA1 | c.2731C>T | p.P871L | 11 | No | - |
|  |  |  |  |  |  | BRCA1 | c.3232A>G | p.E1038G | 11 | No | class 1 |
|  |  |  |  |  |  | BRCA1 | c.3667A>G | p.K1183R | 11 | No | class 1 |
|  |  |  |  |  |  | BRCA1 | c.4427T>C | p.S1436S | 13 | unknown | - |
|  |  |  |  |  |  | BRCA1 | c.4956A>G | p.S1613G | 16 | No | class 1 |
|  |  |  |  |  |  | BRCA2 | c.1342C>A | p.H372N | 10 | No | class 1 |
|  |  |  |  |  |  | BRCA2 | c.[2024T>C] ; [2024T>C] | p.F599S | 10 | unknown | - |
|  |  |  |  |  |  | BRCA2 | c.4035T>C | p.V1269V | 11 | No | - |
|  |  |  |  |  |  | BRCA2 | c.9520T>C | p.Y3098H | 25 | unknown | class 1 |
| SM-41 | Breast cancer | 35 | HBOC | 5 | Germline | BRCA1 | c.2196G>A | p.D693N | 11 | No | class 1 |
|  | Breast cancer | 41 |  |  |  | BRCA1 | c.2201C>T | p.S694S | 11 | unknown | - |
|  |  |  |  |  |  | BRCA1 | c.2430T>C | p.L771L | 11 | unknown | - |
|  |  |  |  |  |  | BRCA1 | c.2731C>T | p.P871L | 11 | No | - |
|  |  |  |  |  |  | BRCA1 | c.3232A>G | p.E1038G | 11 | No | class 1 |
|  |  |  |  |  |  | BRCA1 | c.3667A>G | p.K1183R | 11 | No | class 1 |
|  |  |  |  |  |  | BRCA1 | c.4427T>C | p.S1436S | 13 | unknown | - |
|  |  |  |  |  |  | BRCA1 | c.4956A>G | p.S1613G | 16 | No | class 1 |
|  |  |  |  |  |  | BRCA2 | c.[1342C>A] ; [1342C>A] | p.H372N | 10 | No | class 1 |
|  |  |  |  |  |  | BRCA2 | c.1711G>A | p.A495T | 10 | Unknown | - |
|  |  |  |  |  |  | BRCA2 | c.[2024T>C] ; [2024T>C] | p.F599S | 10 | unknown | - |
|  |  |  |  |  |  | BRCA2 | c.3624A>G | p.K1132K | 11 | No | - |
|  |  |  |  |  |  | BRCA2 | c.4035T>C | p.V1269V | 11 | No | - |
|  |  |  |  |  |  | BRCA2 | c.7470A>G | p.S2414S | 14 | No | - |
| SM-42 | Breast cancer | 60 | HBOC | 4 | Germline | BRCA1 | c.2201C>T | p.S694S | 11 | unknown | - |
|  |  |  |  |  |  | BRCA1 | c.2430T>C | p.L771L | 11 | unknown | - |
|  |  |  |  |  |  | BRCA1 | c.2731C>T | p.P871L | 11 | No | - |
|  |  |  |  |  |  | BRCA1 | c.3232A>G | p.E1038G | 11 | No | class 1 |
|  |  |  |  |  |  | BRCA1 | c.3667A>G | p.K1183R | 11 | No | class 1 |
|  |  |  |  |  |  | BRCA1 | c.4427T>C | p.S1436S | 13 | unknown | - |
|  |  |  |  |  |  | BRCA1 | c.4956A>G | p.S1613G | 16 | No | class 1 |
|  |  |  |  |  |  | BRCA2 | c.203G>A | - | 5' UTR | No | - |
|  |  |  |  |  |  | BRCA2 | c.1342C>A | p.H372N | 10 | No | class 1 |
|  |  |  |  |  |  | BRCA2 | c.[2024T>C] ; [2024T>C] | p.F599S | 10 | unknown | - |
|  |  |  |  |  |  | BRCA2 | c.4035T>C | p.V1269V | 11 | No | - |
|  |  |  |  |  |  | BRCA2 | c.7470A>G | p.S2414S | 14 | No | - |
| SM-43 | Breast cancer | 47 | HBOC | 7 | Germline | BRCA1 | c.2196G>A | p.D693N | 11 | No | class 1 |
|  |  |  |  |  |  | BRCA1 | c.[2201C>T] ; [2201C>T] | p.S694S | 11 | unknown | - |
|  |  |  |  |  |  | BRCA1 | c.[2430T>C]; [2430T>C] | p.L771L | 11 | unknown | - |
|  |  |  |  |  |  | BRCA1 | c.[2731C>T] ; [2731C>T] | p.P871L | 11 | No | - |
|  |  |  |  |  |  | BRCA1 | c.[3232A>G] ; [3232A>G] | p.E1038G | 11 | No | class 1 |
|  |  |  |  |  |  | BRCA1 | c.[3667A>G] ; [3667A>G] | p.K1183R | 11 | No | class 1 |
|  |  |  |  |  |  | BRCA1 | c.[4427T>C]; [4427T>C] | p.S1436S | 13 | unknown | - |
|  |  |  |  |  |  | BRCA1 | c.[4956A>G] ; [4956A>G] | p.S1613G | 16 | No | class 1 |
|  |  |  |  |  |  | BRCA2 | c.[2024T>C] ; [2024T>C] | p.F599S | 10 | unknown | - |
|  |  |  |  |  |  | BRCA2 | c.9313G>A | p.A3029T | 22 | Unknown | - |
| SM-45 | Breast cancer | 74 | HBOC | 9 | Germline | BRCA1 | c.2731C>T | p.P871L | 11 | No | - |
|  |  |  |  |  |  | BRCA2 | c.203G>A | - | 5' UTR | No | - |
|  |  |  |  |  |  | BRCA2 | c.[1342C>A] ; [1342C>A] | p.H372N | 10 | No | class 1 |
|  |  |  |  |  |  | BRCA2 | c.[2024T>C] ; [2024T>C] | p.F599S | 10 | unknown | - |
|  |  |  |  |  |  | BRCA2 | c.3624A>G | p.K1132K | 11 | No | - |
|  |  |  |  |  |  | BRCA2 | c.4035T>C | p.V1269V | 11 | No | - |
|  |  |  |  |  |  | BRCA2 | c.5972C>T | p.T1915M | 11 | Unknown | - |
|  |  |  |  |  |  | BRCA2 | c.7470A>G | p.S2414S | 14 | No | - |
| SM-46 | Breast cancer | 32 | HBOC | 2 | Germline | BRCA1 | c.4158A>G | p.R1347G | 11 | No | class 1 |
|  |  |  |  |  |  | BRCA2 | c.1342C>A | p.H372N | 10 | No | class 1 |
|  |  |  |  |  |  | BRCA2 | c.[2430T>C]; [2430T>C] | p.F599S | 10 | unknown | - |
|  |  |  |  |  |  | BRCA2 | c.4035T>C | p.V1269V | 11 | No | - |
|  |  |  |  |  |  | BRCA2 | c.7697T>C | p.I2490T | 15 | Unknown | - |
|  |  |  |  |  |  | BRCA2 | c.9709A>T | p.K3161* | 25 | Yes | - |
|  |  |  |  |  |  | ATM | Exon 4 deletion | - | 4 | UnkNown | - |
| SM-48 | Breast cancer | 36 | HBOC | 2 | Germline | BRCA1 | c.2196G>A | p.D693N | 11 | No | class 1 |
|  |  |  |  |  |  | BRCA1 | c.2201C>T | p.S694S | 11 | unknown | - |
|  |  |  |  |  |  | BRCA1 | c.2430T>C | p.L771L | 11 | unknown | - |
|  |  |  |  |  |  | BRCA1 | c.2731C>T | p.P871L | 11 | No | - |
|  |  |  |  |  |  | BRCA1 | c.3232A>G | p.E1038G | 11 | No | class 1 |
|  |  |  |  |  |  | BRCA1 | c.3667A>G | p.K1183R | 11 | No | class 1 |
|  |  |  |  |  |  | BRCA1 | c.4427T>C | p.S1436S | 13 | unknown | - |
|  |  |  |  |  |  | BRCA1 | c.4956A>G | p.S1613G | 16 | No | class 1 |
|  |  |  |  |  |  | BRCA2 | c.1093A>C | p.N289H | 10 | No | - |
|  |  |  |  |  |  | BRCA2 | c.1342C>A | p.H372N | 10 | No | class 1 |
|  |  |  |  |  |  | BRCA2 | c.1593A>G | p.S455S | 10 | No | - |
|  |  |  |  |  |  | BRCA2 | c.[2024T>C] ; [2024T>C] | p.F599S | 10 | unknown | - |
|  |  |  |  |  |  | BRCA2 | c.2457T>C | p.H743H | 11 | unknown | - |
|  |  |  |  |  |  | BRCA2 | c.3199A>G | p.N991D | 11 | No | - |
| SM-49 | Melanoma | 29 | HBOC | 2 | Germline | BRCA1 | c.1186A>G | p.Q356R | 11 | Unknown | class 1 |
|  | Breast cancer | 34 |  |  |  | BRCA1 | c.2196G>A | p.D693N | 11 | No | class 1 |
|  |  |  |  |  |  | BRCA1 | c.2201C>T | p.S694S | 11 | unknown | - |
|  |  |  |  |  |  | BRCA1 | c.2430T>C | p.L771L | 11 | unknown | - |
|  |  |  |  |  |  | BRCA1 | c.2731C>T | p.P871L | 11 | No | - |
|  |  |  |  |  |  | BRCA1 | c.3232A>G | p.E1038G | 11 | No | class 1 |
|  |  |  |  |  |  | BRCA1 | c.3667A>G | p.K1183R | 11 | No | class 1 |
|  |  |  |  |  |  | BRCA1 | c.4427T>C | p.S1436S | 13 | unknown | - |
|  |  |  |  |  |  | BRCA1 | c.4956A>G | p.S1613G | 16 | No | class 1 |
|  |  |  |  |  |  | BRCA2 | c.203G>A | - | 5' UTR | No | - |
|  |  |  |  |  |  | BRCA2 | c.[1342C>A] ; [1342C>A] | p.H372N | 10 | No | class 1 |
|  |  |  |  |  |  | BRCA2 | c.[2024T>C] ; [2024T>C] | p.F599S | 10 | unknown | - |
|  |  |  |  |  |  | BRCA2 | c.3624A>G | p.K1132K | 11 | No | - |
|  |  |  |  |  |  | BRCA2 | c.4035T>C | p.V1269V | 11 | No | - |
| SM-50 | Breast cancer | 36 | HBOC | 2 | Germline | BRCA1 | c.2090A>G | p.Q657Q | 11 | No | - |
|  | Breast cancer | 47 |  |  |  | BRCA1 | c.2201C>T | p.S694S | 11 | unknown | - |
|  |  |  |  |  |  | BRCA1 | c.2430T>C | p.L771L | 11 | unknown | - |
|  |  |  |  |  |  | BRCA1 | c.[2731C>T] ; [2731C>T] | p.P871L | 11 | No | - |
|  |  |  |  |  |  | BRCA1 | c.3232A>G | p.E1038G | 11 | No | class 1 |
|  |  |  |  |  |  | BRCA1 | c.3667A>G | p.K1183R | 11 | No | class 1 |
|  |  |  |  |  |  | BRCA1 | c.4427T>C | p.S1436S | 13 | unknown | - |
|  |  |  |  |  |  | BRCA1 | c.4794+1G>A | - | 15 | Yes | - |
|  |  |  |  |  |  | BRCA1 | c.4956A>G | p.S1613G | 16 | No | class 1 |
|  |  |  |  |  |  | BRCA2 | c.[1342C>A] ; [1342C>A] | p.H372N | 10 | No | class 1 |
|  |  |  |  |  |  | BRCA2 | c.[2024T>C] ; [2024T>C] | p.F599S | 10 | unknown | - |
| SM-51 | Breast cancer | 53 | HBOC | 4 | Germline | BRCA1 | c.2201C>T | p.S694S | 11 | unknown | - |
|  | Breast cancer | 63 |  |  |  | BRCA1 | c.2430T>C | p.L771L | 11 | unknown | - |
|  |  |  |  |  |  | BRCA1 | c.2731C>T | p.P871L | 11 | No | - |
|  |  |  |  |  |  | BRCA1 | c.3232A>G | p.E1038G | 11 | No | class 1 |
|  |  |  |  |  |  | BRCA1 | c.3667A>G | p.K1183R | 11 | No | class 1 |
|  |  |  |  |  |  | BRCA1 | c.4427T>C | p.S1436S | 13 | unknown | - |
|  |  |  |  |  |  | BRCA1 | c.4956A>G | p.S1613G | 16 | No | class 1 |
|  |  |  |  |  |  | BRCA2 | c.1342C>A | p.H372N | 10 | No | class 1 |
|  |  |  |  |  |  | BRCA2 | c.[2024T>C] ; [2024T>C] | p.F599S | 10 | unknown | - |
|  |  |  |  |  |  | BRCA2 | c.4035T>C | p.V1269V | 11 | No | - |
|  |  |  |  |  |  | BRCA2 | c.9610C>T | p.R3128* | 25 | Yes | - |
| SM-53 | Breast cancer | 30 | HBOC | 2 | Germline | BRCA1 | c.5002T>C | p.M1628T | 16 | No | - |
|  | Thyroid cancer | 49 |  |  |  | BRCA2 | c.[203G>A] ; [203G>A] | - | 5' UTR | No | - |
|  |  |  |  |  |  | BRCA2 | c.[1342C>A] ; [1342C>A] | p.H372N | 10 | No | class 1 |
|  |  |  |  |  |  | BRCA2 | c.[2024T>C] ; [2024T>C] | p.F599S | 10 | unknown | - |
|  |  |  |  |  |  | BRCA2 | c.[3624A>G]; [3624A>G] | p.K1132K | 11 | No | - |
|  |  |  |  |  |  | BRCA2 | c.6242del4 | p.Asp2005Valfs*34 | 11 | Yes | - |
|  |  |  |  |  |  | BRCA2 | c.[7470A>G] ; [7470A>G] | p.S2414S | 14 | No | - |
| SM-54 | Breast cancer | 56 | HBOC | 4 | Germline | BRCA1 | c.2201C>T | p.S694S | 11 | unknown | - |
|  |  |  |  |  |  | BRCA1 | c.2430T>C | p.L771L | 11 | unknown | - |
|  |  |  |  |  |  | BRCA1 | c.2731C>T | p.P871L | 11 | No | - |
|  |  |  |  |  |  | BRCA1 | c.3232A>G | p.E1038G | 11 | No | class 1 |
|  |  |  |  |  |  | BRCA1 | c.3667A>G | p.K1183R | 11 | No | class 1 |
|  |  |  |  |  |  | BRCA1 | c.4427T>C | p.S1436S | 13 | unknown | - |
|  |  |  |  |  |  | BRCA1 | c.4956A>G | p.S1613G | 16 | No | class 1 |
|  |  |  |  |  |  | BRCA2 | c.203G>A | - | 5' UTR | No | - |
|  |  |  |  |  |  | BRCA2 | c.1342C>A | p.H372N | 10 | No | class 1 |
|  |  |  |  |  |  | BRCA2 | c.[2024T>C] ; [2024T>C] | p.F599S | 10 | unknown | - |
|  |  |  |  |  |  | BRCA2 | c.3624A>G | p.K1132K | 11 | No | - |
|  |  |  |  |  |  | BRCA2 | c.7697T>C | p.I2490T | 15 | Unknown | - |
| SM-55 | Breast cancer | 79 | HBOC | 4 | Germline | BRCA1 | c.1186A>G | p.Q356R | 11 | Unknown | class 1 |
|  | Breast cancer | 81 |  |  |  | BRCA1 | c.2201C>T | p.S694S | 11 | unknown | - |
|  |  |  |  |  |  | BRCA1 | c.2430T>C | p.L771L | 11 | unknown | - |
|  |  |  |  |  |  | BRCA1 | c.2731C>T | p.P871L | 11 | No | - |
|  |  |  |  |  |  | BRCA1 | c.3232A>G | p.E1038G | 11 | No | class 1 |
|  |  |  |  |  |  | BRCA1 | c.3667A>G | p.K1183R | 11 | No | class 1 |
|  |  |  |  |  |  | BRCA1 | c.4427T>C | p.S1436S | 13 | unknown | - |
|  |  |  |  |  |  | BRCA1 | c.4956A>G | p.S1613G | 16 | No | class 1 |
|  |  |  |  |  |  | BRCA2 | c.203G>A | - | 5' UTR | No | - |
|  |  |  |  |  |  | BRCA2 | c.[1342C>A] ; [1342C>A] | p.H372N | 10 | No | class 1 |
|  |  |  |  |  |  | BRCA2 | c.[2024T>C] ; [2024T>C] | p.F599S | 10 | unknown | - |
|  |  |  |  |  |  | BRCA2 | c.3624A>G | p.K1132K | 11 | No | - |
| SM-56 | Breast cancer | 56 | HBOC | 4 | Germline | BRCA1 | c.2201C>T | p.S694S | 11 | unknown | - |
|  |  |  |  |  |  | BRCA1 | c.2430T>C | p.L771L | 11 | unknown | - |
|  |  |  |  |  |  | BRCA1 | c.2731C>T | p.P871L | 11 | No | - |
|  |  |  |  |  |  | BRCA1 | c.3232A>G | p.E1038G | 11 | No | class 1 |
|  |  |  |  |  |  | BRCA1 | c.3667A>G | p.K1183R | 11 | No | class 1 |
|  |  |  |  |  |  | BRCA1 | c.4427T>C | p.S1436S | 13 | unknown | - |
|  |  |  |  |  |  | BRCA1 | c.4956A>G | p.S1613G | 16 | No | class 1 |
|  |  |  |  |  |  | BRCA2 | c.203G>A | - | 5' UTR | No | - |
|  |  |  |  |  |  | BRCA2 | c.1342C>A | p.H372N | 10 | No | class 1 |
|  |  |  |  |  |  | BRCA2 | c.[2024T>C] ; [2024T>C] | p.F599S | 10 | unknown | - |
|  |  |  |  |  |  | BRCA2 | c.3624A>G | p.K1132K | 11 | No | - |
|  |  |  |  |  |  | BRCA2 | c.7470A>G | p.S2414S | 14 | No | - |
| SM-58 | Breast cancer | 68 | HBOC | 4 | Germline | BRCA1 | c.2731C>T | p.P871L | 11 | No | - |
|  |  |  |  |  |  | BRCA2 | c.[203G>A] ; [203G>A] | - | 5' UTR | No | - |
|  |  |  |  |  |  | BRCA2 | c.[1342C>A] ; [1342C>A] | p.H372N | 10 | No | class 1 |
|  |  |  |  |  |  | BRCA2 | c.[2024T>C] ; [2024T>C] | p.F599S | 10 | unknown | - |
|  |  |  |  |  |  | BRCA2 | c.[3624A>G]; [3624A>G] | p.K1132K | 11 | No | - |
|  |  |  |  |  |  | BRCA2 | c.7470A>G | p.S2414S | 14 | No | - |
| SM-59 | Breast cancer | 57 | HBOC | 4 | Germline | BRCA1 | c.2201C>T | p.S694S | 11 | unknown | - |
|  | Colorectal cancer | 57 |  |  |  | BRCA1 | c.2430T>C | p.L771L | 11 | unknown | - |
|  |  |  |  |  |  | BRCA1 | c.2731C>T | p.P871L | 11 | No | - |
|  |  |  |  |  |  | BRCA1 | c.3232A>G | p.E1038G | 11 | No | class 1 |
|  |  |  |  |  |  | BRCA1 | c.3667A>G | p.K1183R | 11 | No | class 1 |
|  |  |  |  |  |  | BRCA1 | c.4427T>C | p.S1436S | 13 | unknown | - |
|  |  |  |  |  |  | BRCA1 | c.4956A>G | p.S1613G | 16 | No | class 1 |
|  |  |  |  |  |  | BRCA2 | c.203G>A | - | 5' UTR | No | - |
|  |  |  |  |  |  | BRCA2 | c.[1342C>A] ; [1342C>A] | p.H372N | 10 | No | class 1 |
|  |  |  |  |  |  | BRCA2 | c.[2024T>C] ; [2024T>C] | p.F599S | 10 | unknown | - |
|  |  |  |  |  |  | BRCA2 | c.3624A>G | p.K1132K | 11 | No | - |
| SM-60 | Breast cancer | 40 | HBOC | 1 | Germline | BRCA1 | c.1186A>G | p.Q356R | 11 | Unknown | class 1 |
|  |  |  |  |  |  | BRCA2 | c.[1342C>A] ; [1342C>A] | p.H372N | 10 | No | class 1 |
|  |  |  |  |  |  | BRCA2 | c.[2024T>C] ; [2024T>C] | p.F599S | 10 | unknown | - |
|  |  |  |  |  |  | BRCA2 | c.[4035T>C] ; [4035T>C] | p.V1269V | 11 | No | - |
| SM-61 | Breast cancer | 51 | HBOC | 4 | Germline | BRCA1 | c.4158A>G | p.R1347G | 11 | No | class 1 |
|  |  |  |  |  |  | BRCA2 | c.1342C>A | p.H372N | 10 | No | class 1 |
|  |  |  |  |  |  | BRCA2 | c.[2024T>C] ; [2024T>C] | p.F599S | 10 | unknown | - |
|  |  |  |  |  |  | BRCA2 | c.4035T>C | p.V1269V | 11 | No | - |
|  |  |  |  |  |  | BRCA2 | c.9709A>T | p.K3161* | 25 | Yes | - |
| SM-62 | Breast cancer | 56 | HBOC | 4 | Germline | BRCA1 | c.1605C>T | p.R496C | 11 | No | class 1 |
|  |  |  |  |  |  | BRCA2 | c.[2024T>C] ; [2024T>C] | p.F599S | 10 | unknown | - |
|  |  |  |  |  |  | PTEN | Exon 2 deletion | - | 2 | Unknown | - |
| SM-64 | Breast cancer | 55 | HBOC | 4 | Germline | BRCA1 | c.2201C>T | p.S694S | 11 | unknown | - |
|  | Colorectal cancer | 57 |  |  |  | BRCA1 | c.2430T>C | p.L771L | 11 | unknown | - |
|  |  |  |  |  |  | BRCA1 | c.2731C>T | p.P871L | 11 | No | - |
|  |  |  |  |  |  | BRCA1 | c.3232A>G | p.E1038G | 11 | No | class 1 |
|  |  |  |  |  |  | BRCA1 | c.3667A>G | p.K1183R | 11 | No | class 1 |
|  |  |  |  |  |  | BRCA1 | c.4427T>C | p.S1436S | 13 | unknown | - |
|  |  |  |  |  |  | BRCA1 | c.4956A>G | p.S1613G | 16 | No | class 1 |
|  |  |  |  |  |  | BRCA2 | c.1093A>C | p.N289H | 10 | No | - |
|  |  |  |  |  |  | BRCA2 | c.1342C>A | p.H372N | 10 | No | class 1 |
|  |  |  |  |  |  | BRCA2 | c.1593A>G | p.S455S | 10 | No | - |
|  |  |  |  |  |  | BRCA2 | c.[2024T>C] ; [2024T>C] | p.F599S | 10 | unknown | - |
|  |  |  |  |  |  | BRCA2 | c.2457T>C | p.H743H | 11 | unknown | - |
|  |  |  |  |  |  | BRCA2 | c.3199A>G | p.N991D | 11 | No | - |
|  |  |  |  |  |  | BRCA2 | c.9079G>A | p.A2951T | 22 | No | - |
| SM-66 | Breast cancer | 35 | HBOC | 6 | Germline | BRCA1 | c.2731C>T | p.P871L | 11 | No | - |
|  | Ovarian cancer | 48 |  |  |  | BRCA2 | c.203G>A | - | 5' UTR | No | - |
|  |  |  |  |  |  | BRCA2 | c.1342C>A | p.H372N | 10 | No | class 1 |
|  |  |  |  |  |  | BRCA2 | c.[2024T>C] ; [2024T>C] | p.F599S | 10 | unknown | - |
|  |  |  |  |  |  | BRCA2 | c.3624A>G | p.K1132K | 11 | No | - |
|  |  |  |  |  |  | BRCA2 | c.7470A>G | p.S2414S | 14 | No | - |
| SM-67 | Breast cancer | 48 | HBOC | 4 | Germline | BRCA1 | c.2201C>T | p.S694S | 11 | unknown | - |
|  |  |  |  |  |  | BRCA1 | c.2430T>C | p.L771L | 11 | unknown | - |
|  |  |  |  |  |  | BRCA1 | c.2731C>T | p.P871L | 11 | No | - |
|  |  |  |  |  |  | BRCA1 | c.3232A>G | p.E1038G | 11 | No | class 1 |
|  |  |  |  |  |  | BRCA1 | c.3667A>G | p.K1183R | 11 | No | class 1 |
|  |  |  |  |  |  | BRCA1 | c.4427T>C | p.S1436S | 13 | unknown | - |
|  |  |  |  |  |  | BRCA1 | c.4956A>G | p.S1613G | 16 | No | class 1 |
|  |  |  |  |  |  | BRCA2 | c.203G>A | - | 5' UTR | No | - |
|  |  |  |  |  |  | BRCA2 | c.[1342C>A] ; [1342C>A] | p.H372N | 10 | No | class 1 |
|  |  |  |  |  |  | BRCA2 | c.[2024T>C] ; [2024T>C] | p.F599S | 10 | unknown | - |
|  |  |  |  |  |  | BRCA2 | c.3624A>G | p.K1132K | 11 | No | - |
|  |  |  |  |  |  | BRCA2 | c.7470A>G | p.S2414S | 14 | No | - |
| SM-68 | Breast cancer | 42 | HBOC | 6 | Germline | BRCA1 | c.2201C>T | p.S694S | 11 | unknown | - |
|  | Ovarian cancer | 52 |  |  |  | BRCA1 | c.2430T>C | p.L771L | 11 | unknown | - |
|  |  |  |  |  |  | BRCA1 | c.2731C>T | p.P871L | 11 | No | - |
|  |  |  |  |  |  | BRCA1 | c.3232A>G | p.E1038G | 11 | No | class 1 |
|  |  |  |  |  |  | BRCA1 | c.3667A>G | p.K1183R | 11 | No | class 1 |
|  |  |  |  |  |  | BRCA1 | c.4427T>C | p.S1436S | 13 | unknown | - |
|  |  |  |  |  |  | BRCA1 | c.4956A>G | p.S1613G | 16 | No | class 1 |
|  |  |  |  |  |  | BRCA2 | c.1342C>A | p.H372N | 10 | No | class 1 |
|  |  |  |  |  |  | BRCA2 | c.[2024T>C] ; [2024T>C] | p.F599S | 10 | unknown | - |
|  |  |  |  |  |  | BRCA2 | c.3624A>G | p.K1132K | 11 | No | - |
|  |  |  |  |  |  | BRCA2 | c.7470A>G | p.S2414S | 14 | No | - |
| SM-69 | Breast cancer | 33 | HBOC | 2 | Germline | BRCA1 | c.710C>T | p.C197C | 9 | No | - |
|  |  |  |  |  |  | BRCA1 | c.5242C>A | p.A1708E | 18 | Yes | class 5 |
|  |  |  |  |  |  | BRCA2 | c.203G>A | - | 5' UTR | No | - |
|  |  |  |  |  |  | BRCA2 | c.1342C>A | p.H372N | 10 | No | class 1 |
|  |  |  |  |  |  | BRCA2 | c.[2024T>C] ; [2024T>C] | p.F599S | 10 | unknown | - |
|  |  |  |  |  |  | BRCA2 | c.3624A>G | p.K1132K | 11 | No | - |
| SM-100 | Colorectal cancer | 56 | HBOC | 4 | Germline | BRCA1 | c.1186A>G | p.Q356R | 11 | Unknown | class 1 |
|  | Breast cancer | 76 |  |  |  | BRCA1 | c.2201C>T | p.S694S | 11 | unknown | - |
|  |  |  |  |  |  | BRCA1 | c.2430T>C | p.L771L | 11 | unknown | - |
|  |  |  |  |  |  | BRCA1 | c.2731C>T | p.P871L | 11 | No | - |
|  |  |  |  |  |  | BRCA1 | c.3232A>G | p.E1038G | 11 | No | class 1 |
|  |  |  |  |  |  | BRCA1 | c.3667A>G | p.K1183R | 11 | No | class 1 |
|  |  |  |  |  |  | BRCA1 | c.4427T>C | p.S1436S | 13 | unknown | - |
|  |  |  |  |  |  | BRCA1 | c.4956A>G | p.S1613G | 16 | No | class 1 |
|  |  |  |  |  |  | BRCA2 | c.203G>A | - | 5' UTR | No | - |
|  |  |  |  |  |  | BRCA2 | c.1342C>A | p.H372N | 10 | No | class 1 |
|  |  |  |  |  |  | BRCA2 | c.[2024T>C] ; [2024T>C] | p.F599S | 10 | unknown | - |
|  |  |  |  |  |  | BRCA2 | c.3624A>G | p.K1132K | 11 | No | - |
|  |  |  |  |  |  | BRCA2 | c.7470A>G | p.S2414S | 14 | No | - |
| SM-72 | Breast cancer | 46 | HBOC | 3 | Germline | BRCA1 | c.2201C>T | p.S694S | 11 | unknown | - |
|  |  |  |  |  |  | BRCA1 | c.2430T>C | p.L771L | 11 | unknown | - |
|  |  |  |  |  |  | BRCA1 | c.2731C>T | p.P871L | 11 | No | - |
|  |  |  |  |  |  | BRCA1 | c.3232A>G | p.E1038G | 11 | No | class 1 |
|  |  |  |  |  |  | BRCA1 | c.3667A>G | p.K1183R | 11 | No | class 1 |
|  |  |  |  |  |  | BRCA1 | c.4427T>C | p.S1436S | 13 | unknown | - |
|  |  |  |  |  |  | BRCA1 | c.4956A>G | p.S1613G | 16 | No | class 1 |
|  |  |  |  |  |  | BRCA2 | c.1342C>A | p.H372N | 10 | No | class 1 |
|  |  |  |  |  |  | BRCA2 | c.[2024T>C] ; [2024T>C] | p.F599S | 10 | unknown | - |
|  |  |  |  |  |  | BRCA2 | c.3624A>G | p.K1132K | 11 | No | - |
|  |  |  |  |  |  | BRCA2 | c.7470A>G | p.S2414S | 14 | No | - |
| SM-73 | Breast cancer | 41 | HBOC | 2 | Germline | BRCA1 | c.2201C>T | p.S694S | 11 | unknown | - |
|  |  |  |  |  |  | BRCA1 | c.2430T>C | p.L771L | 11 | unknown | - |
|  |  |  |  |  |  | BRCA1 | c.2731C>T | p.P871L | 11 | No | - |
|  |  |  |  |  |  | BRCA1 | c.3232A>G | p.E1038G | 11 | No | class 1 |
|  |  |  |  |  |  | BRCA1 | c.3667A>G | p.K1183R | 11 | No | class 1 |
|  |  |  |  |  |  | BRCA1 | c.4427T>C | p.S1436S | 13 | unknown | - |
|  |  |  |  |  |  | BRCA1 | c.4956A>G | p.S1613G | 16 | No | class 1 |
|  |  |  |  |  |  | BRCA2 | c.1342C>A | p.H372N | 10 | No | class 1 |
|  |  |  |  |  |  | BRCA2 | c.[2024T>C] ; [2024T>C] | p.F599S | 10 | unknown | - |
| SM-74 | Hemangiblastoma | 50 | HBOC | 3 | Germline | BRCA1 | c.2080delA | Lys654Serfs*47 | 11 | Yes | - |
|  |  |  |  |  |  | BRCA1 | c.[2201C>T] ; [2201C>T] | p.S694S | 11 | unknown | - |
|  |  |  |  |  |  | BRCA1 | c.[2430T>C]; [2430T>C] | p.L771L | 11 | unknown | - |
|  |  |  |  |  |  | BRCA1 | c.[2731C>T] ; [2731C>T] | p.P871L | 11 | No | - |
|  |  |  |  |  |  | BRCA1 | c.[3232A>G] ; [3232A>G] | p.E1038G | 11 | No | class 1 |
|  |  |  |  |  |  | BRCA1 | c.[3667A>G] ; [3667A>G] | p.K1183R | 11 | No | class 1 |
|  |  |  |  |  |  | BRCA1 | c.[4427T>C]; [4427T>C] | p.S1436S | 13 | unknown | - |
|  |  |  |  |  |  | BRCA1 | c.[4956A>G] ; [4956A>G] | p.S1613G | 16 | No | class 1 |
|  |  |  |  |  |  | BRCA2 | c.1342C>A | p.H372N | 10 | No | class 1 |
|  |  |  |  |  |  | BRCA2 | c.[2024T>C] ; [2024T>C] | p.F599S | 10 | unknown | - |
|  |  |  |  |  |  | BRCA2 | c.4035T>C | p.V1269V | 11 | No | - |
| SM-80 | Breast cancer | 42 | HBOC | 6 | Germline | BRCA1 | c.1446A>T | p.K443* | 11 | Yes | - |
|  | Ovarian cancer | 48 |  |  |  | BRCA1 | c.2201C>T | p.S694S | 11 | unknown | - |
|  |  |  |  |  |  | BRCA1 | c.2430T>C | p.L771L | 11 | unknown | - |
|  |  |  |  |  |  | BRCA1 | c.[2731C>T] ; [2731C>T] | p.P871L | 11 | No | - |
|  |  |  |  |  |  | BRCA1 | c.3232A>G | p.E1038G | 11 | No | class 1 |
|  |  |  |  |  |  | BRCA1 | c.3667A>G | p.K1183R | 11 | No | class 1 |
|  |  |  |  |  |  | BRCA1 | c.4427T>C | p.S1436S | 13 | unknown | - |
|  |  |  |  |  |  | BRCA1 | c.4956A>G | p.S1613G | 16 | No | class 1 |
|  |  |  |  |  |  | BRCA2 | c.203G>A | - | 5' UTR | No | - |
|  |  |  |  |  |  | BRCA2 | c.1324T>G | p.L366V | 10 | Unknown | - |
|  |  |  |  |  |  | BRCA2 | c.[1342C>A] ; [1342C>A] | p.H372N | 10 | No | class 1 |
|  |  |  |  |  |  | BRCA2 | c.[2024T>C] ; [2024T>C] | p.F599S | 10 | unknown | - |
|  |  |  |  |  |  | BRCA2 | c.[3624A>G]; [3624A>G] | p.K1132K | 11 | No | - |
|  |  |  |  |  |  | BRCA2 | c.5972C>T, | p.T1915M |  | Unknown | - |
|  |  |  |  |  |  | BRCA2 | c.[7470A>G] ; [7470A>G] | p.S2414S | 14 | No | - |
| SM-75 | Breast cancer | 50 | HBOC | 3 | Germline | BRCA2 | c.1093A>C | p.N289H | 10 | No | - |
|  |  |  |  |  |  | BRCA2 | c.[1342C>A] ; [1342C>A] | p.H372N | 10 | No | class 1 |
|  |  |  |  |  |  | BRCA2 | c.1593A>G | p.S455S | 10 | No | - |
|  |  |  |  |  |  | BRCA2 | c.[2024T>C] ; [2024T>C] | p.F599S | 10 | unknown | - |
|  |  |  |  |  |  | BRCA2 | c.2457T>C | p.H743H | 11 | unknown | - |
|  |  |  |  |  |  | BRCA2 | c.3199A>G | p.N991D | 11 | No | - |
|  |  |  |  |  |  | BRCA2 | c.4926C>T | p.T1566T | 11 | No | - |
| SM-81 | Fallopian tube | 55 | HBOC | 8 | Germline | BRCA1 | c.2731C>T | p.P871L | 11 | No | - |
|  |  |  |  |  |  | BRCA1 | c.5582insT | His1822Serfs*8 | 23 | Yes | - |
|  |  |  |  |  |  | BRCA2 | c.1342C>A | p.H372N | 10 | No | class 1 |
|  |  |  |  |  |  | BRCA2 | c.[2024T>C] ; [2024T>C] | p.F599S | 10 | unknown | - |
|  |  |  |  |  |  | BRCA2 | c.4909C>A | p.H1561N | 11 | Unknown | - |
|  |  |  |  |  |  | BRCA2 | c.6640G>T | p.V2138F | 11 | Unknown | - |
| SM-82 | Breast cancer | 29 | HBOC | 2 | Germline | BRCA1 | c.2201C>T | p.S694S | 11 | unknown | - |
|  |  |  |  |  |  | BRCA1 | c.2430T>C | p.L771L | 11 | unknown | - |
|  |  |  |  |  |  | BRCA1 | c.2731C>T | p.P871L | 11 | No | - |
|  |  |  |  |  |  | BRCA1 | c.3232A>G | p.E1038G | 11 | No | class 1 |
|  |  |  |  |  |  | BRCA1 | c.3667A>G | p.K1183R | 11 | No | class 1 |
|  |  |  |  |  |  | BRCA1 | c.4427T>C | p.S1436S | 13 | unknown | - |
|  |  |  |  |  |  | BRCA1 | c.4956A>G | p.S1613G | 16 | No | class 1 |
|  |  |  |  |  |  | BRCA2 | c.203G>A | - | 5' UTR | No | - |
|  |  |  |  |  |  | BRCA2 | c.[1342C>A] ; [1342C>A] | p.H372N | 10 | No | class 1 |
|  |  |  |  |  |  | BRCA2 | c.[2024T>C] ; [2024T>C] | p.F599S | 10 | unknown | - |
|  |  |  |  |  |  | BRCA2 | c.3624A>G | p.K1132K | 11 | No | - |
|  |  |  |  |  |  | BRCA2 | c.7470A>G | p.S2414S | 14 | No | - |
|  |  |  |  |  |  | TP53 | c.1010G>A | p.R337H | 10 | Yes | - |
| SM-83 | Breast cancer | 35 | HBOC | 2 | Germline | BRCA2 | c.[2024T>C] ; [2024T>C] | p.F599S | 10 | unknown | - |
| SM-84 | Breast cancer | 35 | HBOC | 2 | Germline | BRCA1 | c.2201C>T | p.S694S | 11 | unknown | - |
|  |  |  |  |  |  | BRCA1 | c.2430T>C | p.L771L | 11 | unknown | - |
|  |  |  |  |  |  | BRCA1 | c.2731C>T | p.P871L | 11 | No | - |
|  |  |  |  |  |  | BRCA1 | c.3232A>G | p.E1038G | 11 | No | class 1 |
|  |  |  |  |  |  | BRCA1 | c.3667A>G | p.K1183R | 11 | No | class 1 |
|  |  |  |  |  |  | BRCA1 | c.4427T>C | p.S1436S | 13 | unknown | - |
|  |  |  |  |  |  | BRCA1 | c.4956A>G | p.S1613G | 16 | No | class 1 |
|  |  |  |  |  |  | BRCA2 | c.203G>A | - | 5' UTR | No | - |
|  |  |  |  |  |  | BRCA2 | c.[1342C>A] ; [1342C>A] | p.H372N | 10 | No | class 1 |
|  |  |  |  |  |  | BRCA2 | c.[2024T>C] ; [2024T>C] | p.F599S | 10 | unknown | - |
|  |  |  |  |  |  | BRCA2 | c.3624A>G | p.K1132K | 11 | No | - |
|  |  |  |  |  |  | BRCA2 | c.4035T>C | p.V1269V | 11 | No | - |
|  |  |  |  |  |  | BRCA2 | c.7470A>G | p.S2414S | 14 | No | - |
|  |  |  |  |  |  | BRCA2 | c.8423T>G | p.L2732* | 18 | Yes | - |
| SM-85 | Breast cancer | 38 | HBOC | 1 | Germline | BRCA1 | c.2731C>T | p.P871L | 11 | No | - |
|  |  |  |  |  |  | BRCA2 | c.[1342C>A] ; [1342C>A] | p.H372N | 10 | No | class 1 |
|  |  |  |  |  |  | BRCA2 | c.[2024T>C] ; [2024T>C] | p.F599S | 10 | unknown | - |
| SM-88 | Breast cancer | 52 | HBOC | 4 | Germline | BRCA1 | c.1186A>G | p.Q356R | 11 | Unknown | class 1 |
|  | Colorectal cancer | 69 |  |  |  | BRCA2 | c.353A>G | p.Y42C | 3 | No | class 1 |
|  |  |  |  |  |  | BRCA2 | c.1342C>A | p.H372N | 10 | No | class 1 |
|  |  |  |  |  |  | BRCA2 | c.[2024T>C] ; [2024T>C] | p.F599S | 10 | unknown | - |
|  |  |  |  |  |  | BRCA2 | c.4035T>C | p.V1269V | 11 | No | - |
|  |  |  |  |  |  | BRCA2 | c.10338G>A | p.R3370R | 27 | No | - |
| SM-87 | Breast cancer | 88 | HBOC | 4 | Germline | BRCA2 | c.203G>A | - | 5' UTR | No | - |
|  | Colorectal cancer | 88 |  |  |  | BRCA2 | c.[1342C>A] ; [1342C>A] | p.H372N | 10 | No | class 1 |
|  |  |  |  |  |  | BRCA2 | c.[2024T>C] ; [2024T>C] | p.F599S | 10 | unknown | - |
|  |  |  |  |  |  | BRCA2 | c.3624A>G | p.K1132K | 11 | No | - |
|  |  |  |  |  |  | BRCA2 | c.4035T>C | p.V1269V | 11 | No | - |
|  |  |  |  |  |  | BRCA2 | c.7470A>G | p.S2414S | 14 | No | - |
| SM-89 | Breast cancer | 40 | HBOC | 2 | Germline | BRCA1 | c.2201C>T | p.S694S | 11 | unknown | - |
|  |  |  |  |  |  | BRCA1 | c.2430T>C | p.L771L | 11 | unknown | - |
|  |  |  |  |  |  | BRCA1 | c.2731C>T | p.P871L | 11 | No | - |
|  |  |  |  |  |  | BRCA1 | c.3232A>G | p.E1038G | 11 | No | class 1 |
|  |  |  |  |  |  | BRCA1 | c.3667A>G | p.K1183R | 11 | No | class 1 |
|  |  |  |  |  |  | BRCA1 | c.4406C>A, | p.Y1429* | 13 | Yes | - |
|  |  |  |  |  |  | BRCA1 | c.4427T>C | p.S1436S | 13 | unknown | - |
|  |  |  |  |  |  | BRCA1 | c.4956A>G | p.S1613G | 16 | No | class 1 |
|  |  |  |  |  |  | BRCA2 | c.[1342C>A] ; [1342C>A] | p.H372N | 10 | No | class 1 |
|  |  |  |  |  |  | BRCA2 | c.[2024T>C] ; [2024T>C] | p.F599S | 10 | unknown | - |
| SM-90 | Breast cancer | 33 | HBOC | 1 | Germline | BRCA1 | c.2201C>T | p.S694S | 11 | unknown | - |
|  |  |  |  |  |  | BRCA1 | c.2430T>C | p.L771L | 11 | unknown | - |
|  |  |  |  |  |  | BRCA1 | c.2731C>T | p.P871L | 11 | No | - |
|  |  |  |  |  |  | BRCA1 | c.3232A>G | p.E1038G | 11 | No | class 1 |
|  |  |  |  |  |  | BRCA1 | c.3667A>G | p.K1183R | 11 | No | class 1 |
|  |  |  |  |  |  | BRCA1 | c.4427T>C | p.S1436S | 13 | unknown | - |
|  |  |  |  |  |  | BRCA1 | c.4956A>G | p.S1613G | 16 | No | class 1 |
|  |  |  |  |  |  | BRCA2 | c.203G>A | - | 5' UTR | No | - |
|  |  |  |  |  |  | BRCA2 | c.[1342C>A] ; [1342C>A] | p.H372N | 10 | No | class 1 |
|  |  |  |  |  |  | BRCA2 | c.[2024T>C] ; [2024T>C] | p.F599S | 10 | unknown | - |
|  |  |  |  |  |  | BRCA2 | c.3624A>G | p.K1132K | 11 | No | - |
|  |  |  |  |  |  | BRCA2 | c.7470A>G | p.S2414S | 14 | No | - |
| SM-91 | Breast cancer | 38 | HBOC | 2 | Germline | BRCA1 | c.2196G>A | p.D693N | 11 | No | class 1 |
|  | Breast cancer | 39 |  |  |  | BRCA1 | c.2201C>T | p.S694S | 11 | unknown | - |
|  |  |  |  |  |  | BRCA1 | c.2430T>C | p.L771L | 11 | unknown | - |
|  |  |  |  |  |  | BRCA1 | c.2731C>T | p.P871L | 11 | No | - |
|  |  |  |  |  |  | BRCA1 | c.3232A>G | p.E1038G | 11 | No | class 1 |
|  |  |  |  |  |  | BRCA1 | c.3667A>G | p.K1183R | 11 | No | class 1 |
|  |  |  |  |  |  | BRCA1 | c.4427T>C | p.S1436S | 13 | unknown | - |
|  |  |  |  |  |  | BRCA1 | c.4956A>G | p.S1613G | 16 | No | class 1 |
|  |  |  |  |  |  | BRCA2 | c.1342C>A | p.H372N | 10 | No | class 1 |
|  |  |  |  |  |  | BRCA2 | c.[2024T>C] ; [2024T>C] | p.F599S | 10 | unknown | - |
|  |  |  |  |  |  | BRCA2 | c.3624A>G | p.K1132K | 11 | No | - |
|  |  |  |  |  |  | BRCA2 | c.7470A>G | p.S2414S | 14 | No | - |
| SM-93 | Breast cancer | 41 | HBOC | 2 | Germline | BRCA1 | c.2201C>T | p.S694S | 11 | unknown | - |
|  |  |  |  |  |  | BRCA1 | c.2430T>C | p.L771L | 11 | unknown | - |
|  |  |  |  |  |  | BRCA1 | c.2731C>T | p.P871L | 11 | No | - |
|  |  |  |  |  |  | BRCA1 | c.3232A>G | p.E1038G | 11 | No | class 1 |
|  |  |  |  |  |  | BRCA1 | c.3667A>G | p.K1183R | 11 | No | class 1 |
|  |  |  |  |  |  | BRCA1 | c.4427T>C | p.S1436S | 13 | unknown | - |
|  |  |  |  |  |  | BRCA1 | c.4956A>G | p.S1613G | 16 | No | class 1 |
|  |  |  |  |  |  | BRCA2 | c.1342C>A | p.H372N | 10 | No | class 1 |
|  |  |  |  |  |  | BRCA2 | c.[2024T>C] ; [2024T>C] | p.F599S | 10 | unknown | - |
|  |  |  |  |  |  | BRCA2 | c.5427C>T | p.S1733S | 11 | No | - |
|  |  |  |  |  |  | BRCA2 | c.9078G>T | p.K2950N | 22 | No | class 1 |
| SM-94 | Breast cancer | 25 | HBOC | 1 | Germline | BRCA2 | c.203G>A | - | 5' UTR | No | - |
|  |  |  |  |  |  | BRCA2 | c.1342C>A | p.H372N | 10 | No | class 1 |
|  |  |  |  |  |  | BRCA2 | c.[2024T>C] ; [2024T>C] | p.F599S | 10 | unknown | - |
|  |  |  |  |  |  | BRCA2 | c.3624A>G | p.K1132K | 11 | No | - |
|  |  |  |  |  |  | BRCA2 | c.7470A>G | p.S2414S | 14 | No | - |
| SM-95 | Breast cancer | 37 | HBOC | 1 | Germline | BRCA1 | c.2201C>T | p.S694S | 11 | unknown | - |
|  |  |  |  |  |  | BRCA1 | c.2430T>C | p.L771L | 11 | unknown | - |
|  |  |  |  |  |  | BRCA1 | c.[2731C>T] ; [2731C>T] | p.P871L | 11 | No | - |
|  |  |  |  |  |  | BRCA1 | c.3232A>G | p.E1038G | 11 | No | class 1 |
|  |  |  |  |  |  | BRCA1 | c.3667A>G | p.K1183R | 11 | No | class 1 |
|  |  |  |  |  |  | BRCA1 | c.4427T>C | p.S1436S | 13 | unknown | - |
|  |  |  |  |  |  | BRCA1 | c.4956A>G | p.S1613G | 16 | No | class 1 |
|  |  |  |  |  |  | BRCA2 | c.1342C>A | p.H372N | 10 | No | class 1 |
|  |  |  |  |  |  | BRCA2 | c.[2024T>C] ; [2024T>C] | p.F599S | 10 | unknown | - |
|  |  |  |  |  |  | BRCA2 | c.3624A>G | p.K1132K | 11 | No | - |
|  |  |  |  |  |  | BRCA2 | c.7470A>G | p.S2414S | 14 | No | - |
| SM-96 | Breast cancer | 41 | HBOC | 2 | Germline | BRCA1 | c.2196G>A | p.D693N | 11 | No | class 1 |
|  |  |  |  |  |  | BRCA1 | c.2201C>T | p.S694S | 11 | unknown | - |
|  |  |  |  |  |  | BRCA1 | c.2430T>C | p.L771L | 11 | unknown | - |
|  |  |  |  |  |  | BRCA1 | c.2731C>T | p.P871L | 11 | No | - |
|  |  |  |  |  |  | BRCA1 | c.3232A>G | p.E1038G | 11 | No | class 1 |
|  |  |  |  |  |  | BRCA1 | c.3667A>G | p.K1183R | 11 | No | class 1 |
|  |  |  |  |  |  | BRCA1 | c.4427T>C | p.S1436S | 13 | unknown | - |
|  |  |  |  |  |  | BRCA1 | c.4956A>G | p.S1613G | 16 | No | class 1 |
|  |  |  |  |  |  | BRCA2 | c.[1342C>A] ; [1342C>A] | p.H372N | 10 | No | class 1 |
|  |  |  |  |  |  | BRCA2 | c.[2024T>C] ; [2024T>C] | p.F599S | 10 | unknown | - |
|  |  |  |  |  |  | BRCA2 | c.4035T>C | p.V1269V | 11 | No | - |
| SM-97 | Linfoma | 27 | HBOC | 3 | Germline | BRCA1 | c.1186A>G | p.Q356R | 11 | Unknown | class 1 |
|  | Breast cancer | 46 |  |  |  | BRCA2 | c.1342C>A | p.H372N | 10 | No | class 1 |
|  |  |  |  |  |  | BRCA2 | c.[2024T>C] ; [2024T>C] | p.F599S | 10 | unknown | - |
|  |  |  |  |  |  | BRCA2 | c.2166C>T | p.S646S | 11 | No | - |
|  |  |  |  |  |  | BRCA2 | c.4035T>C | p.V1269V | 11 | No | - |
|  |  |  |  |  |  | BRCA2 | c.4827A>C | p.K1533N | 11 | Unknown | - |
| SM-98 | Breast cancer | 30 | HBOC | 2 | Germline | BRCA1 | c.2201C>T | p.S694S | 11 | unknown | - |
|  |  |  |  |  |  | BRCA1 | c.2430T>C | p.L771L | 11 | unknown | - |
|  |  |  |  |  |  | BRCA1 | c.2731C>T | p.P871L | 11 | No | - |
|  |  |  |  |  |  | BRCA1 | c.3232A>G | p.E1038G | 11 | No | class 1 |
|  |  |  |  |  |  | BRCA1 | c.3667A>G | p.K1183R | 11 | No | class 1 |
|  |  |  |  |  |  | BRCA1 | c.4427T>C | p.S1436S | 13 | unknown | - |
|  |  |  |  |  |  | BRCA1 | c.4956A>G | p.S1613G | 16 | No | class 1 |
|  |  |  |  |  |  | BRCA2 | c.1342C>A | p.H372N | 10 | No | class 1 |
|  |  |  |  |  |  | BRCA2 | c.[2024T>C] ; [2024T>C] | p.F599S | 10 | unknown | - |
|  |  |  |  |  |  | BRCA2 | c.4035T>C | p.V1269V | 11 | No | - |
| SM-101 | Breast cancer | 44 | HBOC | 2 | Germline | BRCA1 | c.1186A>G | p.Q356R | 11 | Unknown | class 1 |
|  | Melanoma | 51 |  |  |  | BRCA2 | c.203G>A | - | 5' UTR | No | - |
|  | Colorectal cancer | 55 |  |  |  | BRCA2 | c.[1342C>A] ; [1342C>A] | p.H372N | 10 | No | class 1 |
|  |  |  |  |  |  | BRCA2 | c.[2024T>C] ; [2024T>C] | p.F599S | 10 | unknown | - |
|  |  |  |  |  |  | BRCA2 | c.3624A>G | p.K1132K | 11 | No | - |
|  |  |  |  |  |  | BRCA2 | c.4035T>C | p.V1269V | 11 | No | - |
|  |  |  |  |  |  | BRCA2 | c.7470A>G | p.S2414S | 14 | No | - |
| SM-107 | Breast cancer | 61 | HBOC | 4 | Germline | BRCA1 | c.[2201C>T] ; [2201C>T] | p.S694S | 11 | unknown | - |
|  |  |  |  |  |  | BRCA1 | c.[2430T>C]; [2430T>C] | p.L771L | 11 | unknown | - |
|  |  |  |  |  |  | BRCA1 | c.[2731C>T] ; [2731C>T] | p.P871L | 11 | No | - |
|  |  |  |  |  |  | BRCA1 | c.[3232A>G] ; [3232A>G] | p.E1038G | 11 | No | class 1 |
|  |  |  |  |  |  | BRCA1 | c.[3667A>G] ; [3667A>G] | p.K1183R | 11 | No | class 1 |
|  |  |  |  |  |  | BRCA1 | c.[4427T>C]; [4427T>C] | p.S1436S | 13 | unknown | - |
|  |  |  |  |  |  | BRCA1 | c.[4956A>G] ; [4956A>G] | p.S1613G | 16 | No | class 1 |
|  |  |  |  |  |  | BRCA2 | c.1342C>A | p.H372N | 10 | No | class 1 |
|  |  |  |  |  |  | BRCA2 | c.[2024T>C] ; [2024T>C] | p.F599S | 10 | unknown | - |
|  |  |  |  |  |  | BRCA2 | c.3624A>G | p.K1132K | 11 | No | - |
|  |  |  |  |  |  | BRCA2 | c.7470A>G | p.S2414S | 14 | No | - |
| SM-104 | Breast cancer | 32 | HBOC | 2 | Germline | BRCA1 | c.2201C>T | p.S694S | 11 | unknown | - |
|  |  |  |  |  |  | BRCA1 | c.2430T>C | p.L771L | 11 | unknown | - |
|  |  |  |  |  |  | BRCA1 | c.2731C>T | p.P871L | 11 | No | - |
|  |  |  |  |  |  | BRCA1 | c.3232A>G | p.E1038G | 11 | No | class 1 |
|  |  |  |  |  |  | BRCA1 | c.3667A>G | p.K1183R | 11 | No | class 1 |
|  |  |  |  |  |  | BRCA1 | c.4427T>C | p.S1436S | 13 | unknown | - |
|  |  |  |  |  |  | BRCA1 | c.4956A>G | p.S1613G | 16 | No | class 1 |
|  |  |  |  |  |  | BRCA2 | c.[1342C>A] ; [1342C>A] | p.H372N | 10 | No | class 1 |
|  |  |  |  |  |  | BRCA2 | c.[2024T>C] ; [2024T>C] | p.F599S | 10 | unknown | - |
|  |  |  |  |  |  | BRCA2 | c.3034del4 (AAAC) | Ala938Profs*21 | 11 | Yes | - |
| SM-150 | Colorectal cancer | 40 | HBOC | 2 | Germline | BRCA1 | c.1186A>G | p.Q356R | 11 | Unknown | class 1 |
|  | Breast cancer | 43 |  |  |  | BRCA1 | c.2201C>T | p.S694S | 11 | unknown | - |
|  | GIST | 44 |  |  |  | BRCA1 | c.2430T>C | p.L771L | 11 | unknown | - |
|  |  |  |  |  |  | BRCA1 | c.2731C>T | p.P871L | 11 | No | - |
|  |  |  |  |  |  | BRCA1 | c.3232A>G | p.E1038G | 11 | No | class 1 |
|  |  |  |  |  |  | BRCA1 | c.3667A>G | p.K1183R | 11 | No | class 1 |
|  |  |  |  |  |  | BRCA1 | c.4427T>C | p.S1436S | 13 | unknown | - |
|  |  |  |  |  |  | BRCA1 | c.4956A>G | p.S1613G | 16 | No | class 1 |
|  |  |  |  |  |  | BRCA2 | c.1093A>C | p.N289H | 10 | No | - |
|  |  |  |  |  |  | BRCA2 | c.1342C>A | p.H372N | 10 | No | class 1 |
|  |  |  |  |  |  | BRCA2 | c.1593A>G | p.S455S | 10 | No | - |
|  |  |  |  |  |  | BRCA2 | c.[2024T>C] ; [2024T>C] | p.F599S | 10 | unknown | - |
|  |  |  |  |  |  | BRCA2 | c.2457T>C | p.H743H | 11 | unknown | - |
|  |  |  |  |  |  | BRCA2 | c.3199A>G | p.N991D | 11 | No | - |
| MO-03 | Breast cancer | 38 | HBOC | 2 | Germline | BRCA2 | c.1342C>A | p.H372N | 10 | No | class 1 |
|  |  |  |  |  |  | BRCA2 | c.[2024T>C] ; [2024T>C] | p.F599S | 10 | unknown | - |
| MO-07 | Breast cancer | 34 | HBOC | 7 | Germline | BRCA1 | c.5382insC | Gln1756Profs*74 | 20 | Yes | - |
|  |  |  |  |  |  | BRCA2 | c.203G>A | - | 5' UTR | No | - |
|  |  |  |  |  |  | BRCA2 | c.[1342C>A] ; [1342C>A] | p.H372N | 10 | No | class 1 |
|  |  |  |  |  |  | BRCA2 | c.[2024T>C] ; [2024T>C] | p.F599S | 10 | unknown | - |
|  |  |  |  |  |  | BRCA2 | c.3624A>G | p.K1132K | 11 | No | - |
|  |  |  |  |  |  | BRCA2 | c.4035T>C | p.V1269V | 11 | No | - |
|  |  |  |  |  |  | BRCA2 | c.7470A>G | p.S2414S | 14 | No | - |
| MO-09 | Breast cancer | 42 | HBOC | 7 | Germline | BRCA1 | c.2731C>T | p.P871L | 11 | No | - |
|  |  |  |  |  |  | BRCA1 | c.5382insC | - | 20 | Yes | - |
|  |  |  |  |  |  | BRCA2 | c.[1342C>A] ; [1342C>A] | p.H372N | 10 | No | class 1 |
|  |  |  |  |  |  | BRCA2 | c.[2024T>C] ; [2024T>C] | p.F599S | 10 | unknown | - |
|  |  |  |  |  |  | BRCA2 | c.4035T>C | p.V1269V | 11 | No | - |
|  |  |  |  |  |  | BRCA2 | c.6328C>T | p.R2034C | 11 | No | class 1 |
|  |  |  |  |  |  | BRCA2 | c.10462A>G | p.I3412V | 27 | Unknown | - |
| MO-13 | Breast cancer | 32 | HBOC | 2 | Germline | BRCA1 | c.3238G>A | p.S1040N | 11 | unknown | class 1 |
|  | Skin cancer | 51 |  |  |  | BRCA1 | c.4831del5 (TCTCT) | - | 16 | Yes | - |
|  | Ureter cancer | 54 |  |  |  | BRCA2 | c.1342C>A | p.H372N | 10 | No | class 1 |
|  |  |  |  |  |  | BRCA2 | c.[2024T>C] ; [2024T>C] | p.F599S | 10 | unknown | - |
|  |  |  |  |  |  | BRCA2 | c.9058A>T | p.I2944F | 22 | Unknown | - |
| MO-18 | Breast cancer | 44 | HBOC | 2 | Germline | BRCA1 | c.2201C>T | p.S694S | 11 | unknown | - |
|  |  |  |  |  |  | BRCA1 | c.2430T>C | p.L771L | 11 | unknown | - |
|  |  |  |  |  |  | BRCA1 | c.2731C>T | p.P871L | 11 | No | - |
|  |  |  |  |  |  | BRCA1 | c.3232A>G | p.E1038G | 11 | No | class 1 |
|  |  |  |  |  |  | BRCA1 | c.3667A>G | p.K1183R | 11 | No | class 1 |
|  |  |  |  |  |  | BRCA1 | c.4427T>C | p.S1436S | 13 | unknown | - |
|  |  |  |  |  |  | BRCA1 | c.4956A>G | p.S1613G | 16 | No | class 1 |
|  |  |  |  |  |  | BRCA2 | c.1342C>A | p.H372N | 10 | No | class 1 |
|  |  |  |  |  |  | BRCA2 | c.[2024T>C] ; [2024T>C] | p.F599S | 10 | unknown | - |
| MO-17 | Breast cancer | 47 | HBOC | 3 | Germline | BRCA1 | c.1186A>G | p.Q356R | 11 | Unknown | class 1 |
|  |  |  |  |  |  | BRCA2 | c.[1342C>A] ; [1342C>A] | p.H372N | 10 | No | class 1 |
|  |  |  |  |  |  | BRCA2 | c.[2024T>C] ; [2024T>C] | p.F599S | 10 | unknown | - |
|  |  |  |  |  |  | BRCA2 | c.3624A>G | p.K1132K | 11 | No | - |
|  |  |  |  |  |  | BRCA2 | c.7470A>G | p.S2414S | 14 | No | - |
| MO-22 | Breast cancer | 36 | HBOC | 5 | Germline | BRCA1 | c.2196G>A | p.D693N | 11 | No | class 1 |
|  | Breast cancer | 40 |  |  |  | BRCA1 | c.[2201C>T] ; [2201C>T] | p.S694S | 11 | unknown | - |
|  |  |  |  |  |  | BRCA1 | c.[2430T>C]; [2430T>C] | p.L771L | 11 | unknown | - |
|  |  |  |  |  |  | BRCA1 | c.[2731C>T] ; [2731C>T] | p.P871L | 11 | No | - |
|  |  |  |  |  |  | BRCA1 | c.[3232A>G] ; [3232A>G] | p.E1038G | 11 | No | class 1 |
|  |  |  |  |  |  | BRCA1 | c.[3667A>G] ; [3667A>G] | p.K1183R | 11 | No | class 1 |
|  |  |  |  |  |  | BRCA1 | c.[4427T>C]; [4427T>C] | p.S1436S | 13 | unknown | - |
|  |  |  |  |  |  | BRCA1 | c.[4956A>G] ; [4956A>G] | p.S1613G | 16 | No | class 1 |
|  |  |  |  |  |  | BRCA2 | c.[2024T>C] ; [2024T>C] | p.F599S | 10 | unknown | - |
| MO-20 | Breast cancer | 45 | HBOC | 2 | Germline | BRCA1 | c.2201C>T | p.S694S | 11 | unknown | - |
|  |  |  |  |  |  | BRCA1 | c.2430T>C | p.L771L | 11 | unknown | - |
|  |  |  |  |  |  | BRCA1 | c.2731C>T | p.P871L | 11 | No | - |
|  |  |  |  |  |  | BRCA1 | c.3232A>G | p.E1038G | 11 | No | class 1 |
|  |  |  |  |  |  | BRCA1 | c.3667A>G | p.K1183R | 11 | No | class 1 |
|  |  |  |  |  |  | BRCA1 | c.4427T>C | p.S1436S | 13 | unknown | - |
|  |  |  |  |  |  | BRCA1 | c.4956A>G | p.S1613G | 16 | No | class 1 |
|  |  |  |  |  |  | BRCA2 | c.203G>A | - | 5' UTR | No | - |
|  |  |  |  |  |  | BRCA2 | c.[1342C>A] ; [1342C>A] | p.H372N | 10 | No | class 1 |
|  |  |  |  |  |  | BRCA2 | c.[2024T>C] ; [2024T>C] | p.F599S | 10 | unknown | - |
|  |  |  |  |  |  | BRCA2 | c.3624A>G | p.K1132K | 11 | No | - |
|  |  |  |  |  |  | BRCA2 | c.4035T>C | p.V1269V | 11 | No | - |
|  |  |  |  |  |  | BRCA2 | c.7470A>G | p.S2414S | 14 | No | - |
| BT-15 | Breast cancer | 49 | HBOC | 3 | Germline | BRCA1 | c.2731C>T | p.P871L | 11 | No | - |
|  |  |  |  |  |  | BRCA2 | c.203G>A | - | 5' UTR | No | - |
|  |  |  |  |  |  | BRCA2 | c.1342C>A | p.H372N | 10 | No | class 1 |
|  |  |  |  |  |  | BRCA2 | c.[2024T>C] ; [2024T>C] | p.F599S | 10 | unknown | - |
|  |  |  |  |  |  | BRCA2 | c.3624A>G | p.K1132K | 11 | No | - |
| MO-15 | Breast cancer | 43 | HBOC | 6 | Germline | BRCA1 | c.560+2T>A | p.Ser127Thrfs*11 | i7 | Yes | - |
|  | Ovarian cancer | 56 |  |  |  | BRCA2 | c.1342C>A | p.H372N | 10 | No | class 1 |
|  |  |  |  |  |  | BRCA2 | c.[2024T>C] ; [2024T>C] | p.F599S | 10 | unknown | - |
| MO-06 | Breast cancer | 36 | HBOC | 1 | Germline | BRCA2 | c.203G>A | - | 5' UTR | No | - |
|  |  |  |  |  |  | BRCA2 | c.1342C>A | p.H372N | 10 | No | class 1 |
|  |  |  |  |  |  | BRCA2 | c.[2024T>C] ; [2024T>C] | p.F599S | 10 | unknown | - |
|  |  |  |  |  |  | BRCA2 | c.3624A>G | p.K1132K | 11 | No | - |
|  |  |  |  |  |  | BRCA2 | c.7470A>G | p.S2414S | 14 | No | - |
| MO-23 | Breast cancer | 50 | HBOC | 3 | Germline | BRCA2 | c.1093A>C | p.N289H | 10 | No | - |
|  |  |  |  |  |  | BRCA2 | c.[1342C>A] ; [1342C>A] | p.H372N | 10 | No | class 1 |
|  |  |  |  |  |  | BRCA2 | c.1593A>G | p.S455S | 10 | No | - |
|  |  |  |  |  |  | BRCA2 | c.[2024T>C] ; [2024T>C] | p.F599S | 10 | unknown | - |
|  |  |  |  |  |  | BRCA2 | c.2457T>C | p.H743H | 11 | unknown | - |
|  |  |  |  |  |  | BRCA2 | c.3199A>G | p.N991D | 11 | No | - |
| SM-165 | Breast cancer | 27 | HBOC | 1 | Germline | BRCA1 | c.2201C>T | p.S694S | 11 | unknown | - |
|  |  |  |  |  |  | BRCA1 | c.2430T>C | p.L771L | 11 | unknown | - |
|  |  |  |  |  |  | BRCA1 | c.2731C>T | p.P871L | 11 | No | - |
|  |  |  |  |  |  | BRCA1 | c.3232A>G | p.E1038G | 11 | No | class 1 |
|  |  |  |  |  |  | BRCA1 | c.3667A>G | p.K1183R | 11 | No | class 1 |
|  |  |  |  |  |  | BRCA1 | c.4427T>C | p.S1436S | 13 | unknown | - |
|  |  |  |  |  |  | BRCA1 | c.4956A>G | p.S1613G | 16 | No | class 1 |
|  |  |  |  |  |  | BRCA2 | c.1342C>A | p.H372N | 10 | No | class 1 |
|  |  |  |  |  |  | BRCA2 | c.[2024T>C] ; [2024T>C] | p.F599S | 10 | unknown | - |
| MO-28 | Breast cancer | 48 | HBOC | 3 | Germline | BRCA1 | c.2201C>T | p.S694S | 11 | unknown | - |
|  |  |  |  |  |  | BRCA1 | c.2430T>C | p.L771L | 11 | unknown | - |
|  |  |  |  |  |  | BRCA1 | c.2731C>T | p.P871L | 11 | No | - |
|  |  |  |  |  |  | BRCA1 | c.3232A>G | p.E1038G | 11 | No | class 1 |
|  |  |  |  |  |  | BRCA1 | c.3238G>A | p.S1040N | 11 | unknown | class 1 |
|  |  |  |  |  |  | BRCA1 | c.3667A>G | p.K1183R | 11 | No | class 1 |
|  |  |  |  |  |  | BRCA1 | c.4427T>C | p.S1436S | 13 | unknown | - |
|  |  |  |  |  |  | BRCA1 | c.4956A>G | p.S1613G | 16 | No | class 1 |
|  |  |  |  |  |  | BRCA1 | Exon 24 dupication | - | 24 | Yes | - |
|  |  |  |  |  |  | BRCA2 | c.203G>A | - | 5' UTR | No | - |
|  |  |  |  |  |  | BRCA2 | c.[1342C>A] ; [1342C>A] | p.H372N | 10 | No | class 1 |
|  |  |  |  |  |  | BRCA2 | c.[2024T>C] ; [2024T>C] | p.F599S | 10 | unknown | - |
|  |  |  |  |  |  | BRCA2 | c.3624A>G | p.K1132K | 11 | No | - |
|  |  |  |  |  |  | BRCA2 | c.4035T>C | p.V1269V | 11 | No | - |
|  |  |  |  |  |  | BRCA2 | c.4296G>A, | p.L1356L | 11 | No | - |
| MO-27 | Breast cancer | 28 | HBOC | 2 | Germline | BRCA1 | c.2196G>A | p.D693N | 11 | No | class 1 |
|  |  |  |  |  |  | BRCA1 | c.2201C>T | p.S694S | 11 | unknown | - |
|  |  |  |  |  |  | BRCA1 | c.2430T>C | p.L771L | 11 | unknown | - |
|  |  |  |  |  |  | BRCA1 | c.2731C>T | p.P871L | 11 | No | - |
|  |  |  |  |  |  | BRCA1 | c.3232A>G | p.E1038G | 11 | No | class 1 |
|  |  |  |  |  |  | BRCA1 | c.3667A>G | p.K1183R | 11 | No | class 1 |
|  |  |  |  |  |  | BRCA1 | c.4427T>C | p.S1436S | 13 | unknown | - |
|  |  |  |  |  |  | BRCA1 | c.4956A>G | p.S1613G | 16 | No | class 1 |
|  |  |  |  |  |  | BRCA2 | c.1342C>A | p.H372N | 10 | No | class 1 |
|  |  |  |  |  |  | BRCA2 | c.[2024T>C] ; [2024T>C] | p.F599S | 10 | unknown | - |
|  |  |  |  |  |  | BRCA2 | c.10204A>T | p.K3326* | 27 | No | class 1 |
| MO-25 | Breast cancer | 39 | HBOC | 2 | Germline | BRCA1 | c.2201C>T | p.S694S | 11 | unknown | - |
|  |  |  |  |  |  | BRCA1 | c.2430T>C | p.L771L | 11 | unknown | - |
|  |  |  |  |  |  | BRCA1 | c.[2731C>T] ; [2731C>T] | p.P871L | 11 | No | - |
|  |  |  |  |  |  | BRCA1 | c.3232A>G | p.E1038G | 11 | No | class 1 |
|  |  |  |  |  |  | BRCA1 | c.3238G>A | p.S1040N | 11 | unknown | class 1 |
|  |  |  |  |  |  | BRCA1 | c.3667A>G | p.K1183R | 11 | No | class 1 |
|  |  |  |  |  |  | BRCA1 | c.4427T>C | p.S1436S | 13 | unknown | - |
|  |  |  |  |  |  | BRCA1 | c.4956A>G | p.S1613G | 16 | No | class 1 |
|  |  |  |  |  |  | BRCA2 | c.[1342C>A] ; [1342C>A] | p.H372N | 10 | No | class 1 |
|  |  |  |  |  |  | BRCA2 | c.[2024T>C] ; [2024T>C] | p.F599S | 10 | unknown | - |
| MO-26 | Breast cancer | 40 | HBOC | 2 | Germline | BRCA1 | c.1499insA | Phe461Ilefs*19 | 11 | Yes | - |
|  |  |  |  |  |  | BRCA1 | c.2201C>T | p.S694S | 11 | unknown | - |
|  |  |  |  |  |  | BRCA1 | c.2430T>C | p.L771L | 11 | unknown | - |
|  |  |  |  |  |  | BRCA1 | c.2731C>T | p.P871L | 11 | No | - |
|  |  |  |  |  |  | BRCA1 | c.3232A>G | p.E1038G | 11 | No | class 1 |
|  |  |  |  |  |  | BRCA1 | c.3667A>G | p.K1183R | 11 | No | class 1 |
|  |  |  |  |  |  | BRCA1 | c.4427T>C | p.S1436S | 13 | unknown | - |
|  |  |  |  |  |  | BRCA1 | c.4956A>G | p.S1613G | 16 | No | class 1 |
|  |  |  |  |  |  | BRCA2 | c.[1342C>A] ; [1342C>A] | p.H372N | 10 | No | class 1 |
|  |  |  |  |  |  | BRCA2 | c.[2024T>C] ; [2024T>C] | p.F599S | 10 | unknown | - |
|  |  |  |  |  |  | BRCA2 | c.4035T>C | p.V1269V | 11 | No | - |
| LFS-211 | Breast cancer | 22 | HBOC | 2 | Germline | BRCA1 | c.2201C>T | p.S694S | 11 | unknown | - |
|  |  |  |  |  |  | BRCA1 | c.2430T>C | p.L771L | 11 | unknown | - |
|  |  |  |  |  |  | BRCA1 | c.2731C>T | p.P871L | 11 | No | - |
|  |  |  |  |  |  | BRCA1 | c.3232A>G | p.E1038G | 11 | No | class 1 |
|  |  |  |  |  |  | BRCA1 | c.3667A>G | p.K1183R | 11 | No | class 1 |
|  |  |  |  |  |  | BRCA1 | c.4956A>G | p.S1613G | 16 | No | class 1 |
|  |  |  |  |  |  | BRCA2 | c.203G>A | - | 5' UTR | No | - |
|  |  |  |  |  |  | BRCA2 | c.[1342C>A] ; [1342C>A] | p.H372N | 10 | No | class 1 |
|  |  |  |  |  |  | BRCA2 | c.[2024T>C] ; [2024T>C] | p.F599S | 10 | unknown | - |
|  |  |  |  |  |  | BRCA2 | c.3624A>G | p.K1132K | 11 | No | - |
|  |  |  |  |  |  | BRCA2 | c.4035T>C | p.V1269V | 11 | No | - |
| MO-29 | Breast cancer | 40 | HBOC | 2 | Germline | BRCA2 | c.203G>A | - | 5' UTR | No | - |
|  |  |  |  |  |  | BRCA2 | c.1342C>A | p.H372N | 10 | No | class 1 |
|  |  |  |  |  |  | BRCA2 | c.[2024T>C] ; [2024T>C] | p.F599S | 10 | unknown | - |
|  |  |  |  |  |  | BRCA2 | c.3624A>G | p.K1132K | 11 | No | - |
|  |  |  |  |  |  | BRCA2 | c.7470A>G | p.S2414S | 14 | No | - |
| SM-173 | Breast cancer | 38 | HBOC | 1 | Germline | BRCA1 | c.1186A>G | p.Q356R | 11 | Unknown | class 1 |
|  |  |  |  |  |  | BRCA2 | c.[2024T>C] ; [2024T>C] | p.F599S | 10 | unknown | - |
| MO-30 | Breast cancer | 40 | HBOC | 2 | Germline | BRCA2 | c.[2024T>C] ; [2024T>C] | p.F599S | 10 | unknown | - |
|  |  |  |  |  |  | BRCA2 | c.7697T>C | p.I2490T | 15 | Unknown | - |
| MO-33 |  |  | HBOC | 2 | Germline | BRCA1 | c.1186A>G | p.Q356R | 11 | Unknown | class 1 |
|  |  |  |  |  |  | BRCA2 | c.[1342C>A] ; [1342C>A] | p.H372N | 10 | No | class 1 |
|  |  |  |  |  |  | BRCA2 | c.[2024T>C] ; [2024T>C] | p.F599S | 10 | unknown | - |
|  |  |  |  |  |  | BRCA2 | c.4035T>C | p.V1269V | 11 | No | - |
| MO-34 | Bilateral Breast cancer | 38 | HBOC | 5 | Germline | BRCA1 | c.1186A>G | p.Q356R | 11 | Unknown | class 1 |
|  |  |  |  |  |  | BRCA2 | c.1342C>A | p.H372N | 10 | No | class 1 |
|  |  |  |  |  |  | BRCA2 | c.[2024T>C] ; [2024T>C] | p.F599S | 10 | unknown | - |
|  |  |  |  |  |  | BRCA2 | c.4035T>C | p.V1269V | 11 | No | - |
| MO-36 | Breast cancer | 35 | HBOC | 2 | Germline | BRCA1 | c.2201C>T | p.S694S | 11 | unknown | - |
|  |  |  |  |  |  | BRCA1 | c.2430T>C | p.L771L | 11 | unknown | - |
|  |  |  |  |  |  | BRCA1 | c.2731C>T | p.P871L | 11 | No | - |
|  |  |  |  |  |  | BRCA1 | c.3232A>G | p.E1038G | 11 | No | class 1 |
|  |  |  |  |  |  | BRCA1 | c.3667A>G | p.K1183R | 11 | No | class 1 |
|  |  |  |  |  |  | BRCA1 | c.4427T>C | p.S1436S | 13 | unknown | - |
|  |  |  |  |  |  | BRCA1 | c.4956A>G | p.S1613G | 16 | No | class 1 |
|  |  |  |  |  |  | BRCA2 | c.203G>A | - | 5' UTR | No | - |
|  |  |  |  |  |  | BRCA2 | c.1342C>A | p.H372N | 10 | No | class 1 |
|  |  |  |  |  |  | BRCA2 | c.[2024T>C] ; [2024T>C] | p.F599S | 10 | unknown | - |
|  |  |  |  |  |  | BRCA2 | c.3624A>G | p.K1132K | 11 | No | - |
|  |  |  |  |  |  | BRCA2 | c.7470A>G | p.S2414S | 14 | No | - |
| MO-38 | Breast cancer | 42 | HBOC | 5 | Germline | BRCA1 | c.307T>A | p.L63* | 5 | Yes | - |
|  | Breast cancer | 44 |  |  |  | BRCA1 | c.[2201C>T] ; [2201C>T] | p.S694S | 11 | unknown | - |
|  |  |  |  |  |  | BRCA1 | c.[2430T>C]; [2430T>C] | p.L771L | 11 | unknown | - |
|  |  |  |  |  |  | BRCA1 | c.[2731C>T] ; [2731C>T] | p.P871L | 11 | No | - |
|  |  |  |  |  |  | BRCA1 | c.[3232A>G] ; [3232A>G] | p.E1038G | 11 | No | class 1 |
|  |  |  |  |  |  | BRCA1 | c.[3667A>G] ; [3667A>G] | p.K1183R | 11 | No | class 1 |
|  |  |  |  |  |  | BRCA1 | c.[4427T>C]; [4427T>C] | p.S1436S | 13 | unknown | - |
|  |  |  |  |  |  | BRCA1 | c.[4956A>G] ; [4956A>G] | p.S1613G | 16 | No | class 1 |
|  |  |  |  |  |  | BRCA2 | c.1342C>A | p.H372N | 10 | No | class 1 |
|  |  |  |  |  |  | BRCA2 | c.[2024T>C] ; [2024T>C] | p.F599S | 10 | unknown | - |
|  |  |  |  |  |  | BRCA2 | c.4035T>C | p.V1269V | 11 | No | - |
| MO-40 |  |  | HBOC | 2 | Germline | BRCA1 | c.233G>A | p.K38K | 3 | No | - |
|  |  |  |  |  |  | BRCA2 | c.203G>A | - | 5' UTR | No | - |
|  |  |  |  |  |  | BRCA2 | c.[1342C>A] ; [1342C>A] | p.H372N | 10 | No | class 1 |
|  |  |  |  |  |  | BRCA2 | c.[2024T>C] ; [2024T>C] | p.F599S | 10 | unknown | - |
|  |  |  |  |  |  | BRCA2 | c.2578A>G | p.M784V | 11 | Unknown | class 3 |
|  |  |  |  |  |  | BRCA2 | c.3624A>G | p.K1132K | 11 | No | - |
|  |  |  |  |  |  | BRCA2 | c.4035T>C | p.V1269V | 11 | No | - |
|  |  |  |  |  |  | BRCA2 | c.7470A>G | p.S2414S | 14 | No | - |
| MO-41 | Lobular Breast cancer | 47 | HBOC | 3 | Germline | BRCA1 | c.2201C>T | p.S694S | 11 | unknown | - |
|  |  |  |  |  |  | BRCA1 | c.2430T>C | p.L771L | 11 | unknown | - |
|  |  |  |  |  |  | BRCA1 | c.2731C>T | p.P871L | 11 | No | - |
|  |  |  |  |  |  | BRCA1 | c.3232A>G | p.E1038G | 11 | No | class 1 |
|  |  |  |  |  |  | BRCA1 | c.3238G>A | p.S1040N | 11 | unknown | class 1 |
|  |  |  |  |  |  | BRCA1 | c.3667A>G | p.K1183R | 11 | No | class 1 |
|  |  |  |  |  |  | BRCA1 | c.4427T>C | p.S1436S | 13 | unknown | - |
|  |  |  |  |  |  | BRCA1 | c.4956A>G | p.S1613G | 16 | No | class 1 |
|  |  |  |  |  |  | BRCA2 | c.203G>A | - | 5' UTR | No | - |
|  |  |  |  |  |  | BRCA2 | c.[1342C>A] ; [1342C>A] | p.H372N | 10 | No | class 1 |
|  |  |  |  |  |  | BRCA2 | c.[2024T>C] ; [2024T>C] | p.F599S | 10 | unknown | - |
|  |  |  |  |  |  | BRCA2 | c.3624A>G | p.K1132K | 11 | No | - |
|  |  |  |  |  |  | BRCA2 | c.7470A>G | p.S2414S | 14 | No | - |
|  |  |  |  |  |  | CHEK2 | 1100delC | - |  | Yes | - |
| MO-44 | Breast cancer | 60 | HBOC | 4 | Germline | BRCA1 | c.2196G>A | p.D693N | 11 | No | class 1 |
|  |  |  |  |  |  | BRCA1 | c.2201C>T | p.S694S | 11 | unknown | - |
|  |  |  |  |  |  | BRCA1 | c.2430T>C | p.L771L | 11 | unknown | - |
|  |  |  |  |  |  | BRCA1 | c.2731C>T | p.P871L | 11 | No | - |
|  |  |  |  |  |  | BRCA1 | c.3232A>G | p.E1038G | 11 | No | class 1 |
|  |  |  |  |  |  | BRCA1 | c.3238G>A | p.S1040N | 11 | unknown | class 1 |
|  |  |  |  |  |  | BRCA1 | c.3667A>G | p.K1183R | 11 | No | class 1 |
|  |  |  |  |  |  | BRCA1 | c.4427T>C | p.S1436S | 13 | unknown | - |
|  |  |  |  |  |  | BRCA1 | c.4956A>G | p.S1613G | 16 | No | class 1 |
|  |  |  |  |  |  | BRCA2 | c.203G>A | - | 5' UTR | No | - |
|  |  |  |  |  |  | BRCA2 | c.1342C>A | p.H372N | 10 | No | class 1 |
|  |  |  |  |  |  | BRCA2 | c.[2024T>C] ; [2024T>C] | p.F599S | 10 | unknown | - |
|  |  |  |  |  |  | BRCA2 | c.3624A>G | p.K1132K | 11 | No | - |
|  |  |  |  |  |  | BRCA2 | c.7470A>G | p.S2414S | 14 | No | - |
| MO-45 | Breast cancer | 48 | HBOC | 7 | Germline | BRCA1 | c.2201C>T | p.S694S | 11 | unknown | - |
|  |  |  |  |  |  | BRCA1 | c.2430T>C | p.L771L | 11 | unknown | - |
|  |  |  |  |  |  | BRCA1 | c.2731C>T | p.P871L | 11 | No | - |
|  |  |  |  |  |  | BRCA1 | c.3232A>G | p.E1038G | 11 | No | class 1 |
|  |  |  |  |  |  | BRCA1 | c.3667A>G | p.K1183R | 11 | No | class 1 |
|  |  |  |  |  |  | BRCA1 | c.4427T>C | p.S1436S | 13 | unknown | - |
|  |  |  |  |  |  | BRCA1 | c.4956A>G | p.S1613G | 16 | No | class 1 |
|  |  |  |  |  |  | BRCA1 | c.5382insC | p.Gln1756Profs*74 | 20 | Yes | - |
|  |  |  |  |  |  | BRCA2 | c.[2024T>C] ; [2024T>C] | p.F599S | 10 | unknown | - |
| MO-46 |  |  | HBOC | 3 | Germline | BRCA2 | c.203G>A | - | 5' UTR | No | - |
|  |  |  |  |  |  | BRCA2 | c.[1342C>A] ; [1342C>A] | p.H372N | 10 | No | class 1 |
|  |  |  |  |  |  | BRCA2 | c.[2024T>C] ; [2024T>C] | p.F599S | 10 | unknown | - |
|  |  |  |  |  |  | BRCA2 | c.3624A>G | p.K1132K | 11 | No | - |
|  |  |  |  |  |  | BRCA2 | c.7470A>G | p.S2414S | 14 | No | - |
| MO-31 | Breast cancer | 38 | HBOC | 2 | Germline | BRCA1 | c.5563G>A | p.W1815* | 23 | Yes | - |
|  | Peritoneal cancer | 63 |  |  |  | BRCA2 | c.1093A>C | p.N289H | 10 | No | - |
|  |  |  |  |  |  | BRCA2 | c.[1342C>A] ; [1342C>A] | p.H372N | 10 | No | class 1 |
|  |  |  |  |  |  | BRCA2 | c.1593A>G | p.S455S | 10 | No | - |
|  |  |  |  |  |  | BRCA2 | c.[2024T>C] ; [2024T>C] | p.F599S | 10 | unknown | - |
|  |  |  |  |  |  | BRCA2 | c.2457T>C | p.H743H | 11 | unknown | - |
|  |  |  |  |  |  | BRCA2 | c.3199A>G | p.N991D | 11 | No | - |
| MO-35 | Ovarian cancer | 49 | HBOC | 6 | Germline | BRCA1 | c.[2201C>T] ; [2201C>T] | p.S694S | 11 | unknown | - |
|  | Breast cancer | 50 |  |  |  | BRCA1 | c.[2430T>C]; [2430T>C] | p.L771L | 11 | unknown | - |
|  |  |  |  |  |  | BRCA1 | c.[2731C>T] ; [2731C>T] | p.P871L | 11 | No | - |
|  |  |  |  |  |  | BRCA1 | c.[3232A>G] ; [3232A>G] | p.E1038G | 11 | No | class 1 |
|  |  |  |  |  |  | BRCA1 | c.[3667A>G] ; [3667A>G] | p.K1183R | 11 | No | class 1 |
|  |  |  |  |  |  | BRCA1 | c.[4427T>C]; [4427T>C] | p.S1436S | 13 | unknown | - |
|  |  |  |  |  |  | BRCA1 | c.[4956A>G] ; [4956A>G] | p.S1613G | 16 | No | class 1 |
|  |  |  |  |  |  | BRCA2 | c.[1342C>A] ; [1342C>A] | p.H372N | 10 | No | class 1 |
|  |  |  |  |  |  | BRCA2 | c.[2024T>C] ; [2024T>C] | p.F599S | 10 | unknown | - |
|  |  |  |  |  |  | BRCA2 | c.4035T>C | p.V1269V | 11 | No | - |
| MO-37 |  |  | HBOC | 2 | Germline | BRCA1 | c.2201C>T | p.S694S | 11 | unknown | - |
|  |  |  |  |  |  | BRCA1 | c.2430T>C | p.L771L | 11 | unknown | - |
|  |  |  |  |  |  | BRCA1 | c.2731C>T | p.P871L | 11 | No | - |
|  |  |  |  |  |  | BRCA1 | c.3232A>G | p.E1038G | 11 | No | class 1 |
|  |  |  |  |  |  | BRCA1 | c.3667A>G | p.K1183R | 11 | No | class 1 |
|  |  |  |  |  |  | BRCA1 | c.3759G>T | p.E1214* | 11 | Yes | - |
|  |  |  |  |  |  | BRCA1 | c.4427T>C | p.S1436S | 13 | unknown | - |
|  |  |  |  |  |  | BRCA1 | c.4956A>G | p.S1613G | 16 | No | class 1 |
|  |  |  |  |  |  | BRCA2 | c.1342C>A | p.H372N | 10 | No | class 1 |
|  |  |  |  |  |  | BRCA2 | c.[2024T>C] ; [2024T>C] | p.F599S | 10 | unknown | - |
|  |  |  |  |  |  | BRCA2 | c.4035T>C | p.V1269V | 11 | No | - |
|  |  |  |  |  |  | BRCA2 | c.6359G>T | p.G2044V | 11 | Unknown | - |
| SM-99 | Breast cancer | 58 | HBOC | 4 | Germline | BRCA1 | c.2201C>T | p.S694S | 11 | unknown | - |
|  |  |  |  |  |  | BRCA1 | c.2430T>C | p.L771L | 11 | unknown | - |
|  |  |  |  |  |  | BRCA1 | c.2731C>T | p.P871L | 11 | No | - |
|  |  |  |  |  |  | BRCA1 | c.3232A>G | p.E1038G | 11 | No | class 1 |
|  |  |  |  |  |  | BRCA1 | c.3667A>G | p.K1183R | 11 | No | class 1 |
|  |  |  |  |  |  | BRCA1 | c.4427T>C | p.S1436S | 13 | unknown | - |
|  |  |  |  |  |  | BRCA1 | c.4956A>G | p.S1613G | 16 | No | class 1 |
|  |  |  |  |  |  | BRCA2 | c.[1342C>A] ; [1342C>A] | p.H372N | 10 | No | class 1 |
|  |  |  |  |  |  | BRCA2 | c.[2024T>C] ; [2024T>C] | p.F599S | 10 | unknown | - |
|  |  |  |  |  |  | BRCA2 | c.[4035T>C] ; [4035T>C] | p.V1269V | 11 | No | - |
| SM-196 | Breast cancer | 46 | HBOC | 3 | Germline | BRCA1 | c.2196G>A | p.D693N | 11 | No | class 1 |
|  |  |  |  |  |  | BRCA1 | c.2201C>T | p.S694S | 11 | unknown | - |
|  |  |  |  |  |  | BRCA1 | c.2430T>C | p.L771L | 11 | unknown | - |
|  |  |  |  |  |  | BRCA1 | c.2731C>T | p.P871L | 11 | No | - |
|  |  |  |  |  |  | BRCA1 | c.3232A>G | p.E1038G | 11 | No | class 1 |
|  |  |  |  |  |  | BRCA1 | c.3667A>G | p.K1183R | 11 | No | class 1 |
|  |  |  |  |  |  | BRCA1 | c.4427T>C | p.S1436S | 13 | unknown | - |
|  |  |  |  |  |  | BRCA1 | c.4956A>G | p.S1613G | 16 | No | class 1 |
|  |  |  |  |  |  | BRCA2 | c.1342C>A | p.H372N | 10 | No | class 1 |
|  |  |  |  |  |  | BRCA2 | c.2024T>C | p.F599S | 10 | unknown | - |
|  |  |  |  |  |  | BRCA2 | c.4035T>C | p.V1269V | 11 | No | - |
