# Supplementary figures and images for: Hereditary breast and ovarian cancer: assessment of point mutations and copy number variations in Brazilian patients
Source: BMC Med Genet. 2014 May 15;15:55. doi: 10.1186/1471-2350-15-55 (PMC4038072; doi:10.1186/1471-2350-15-55)

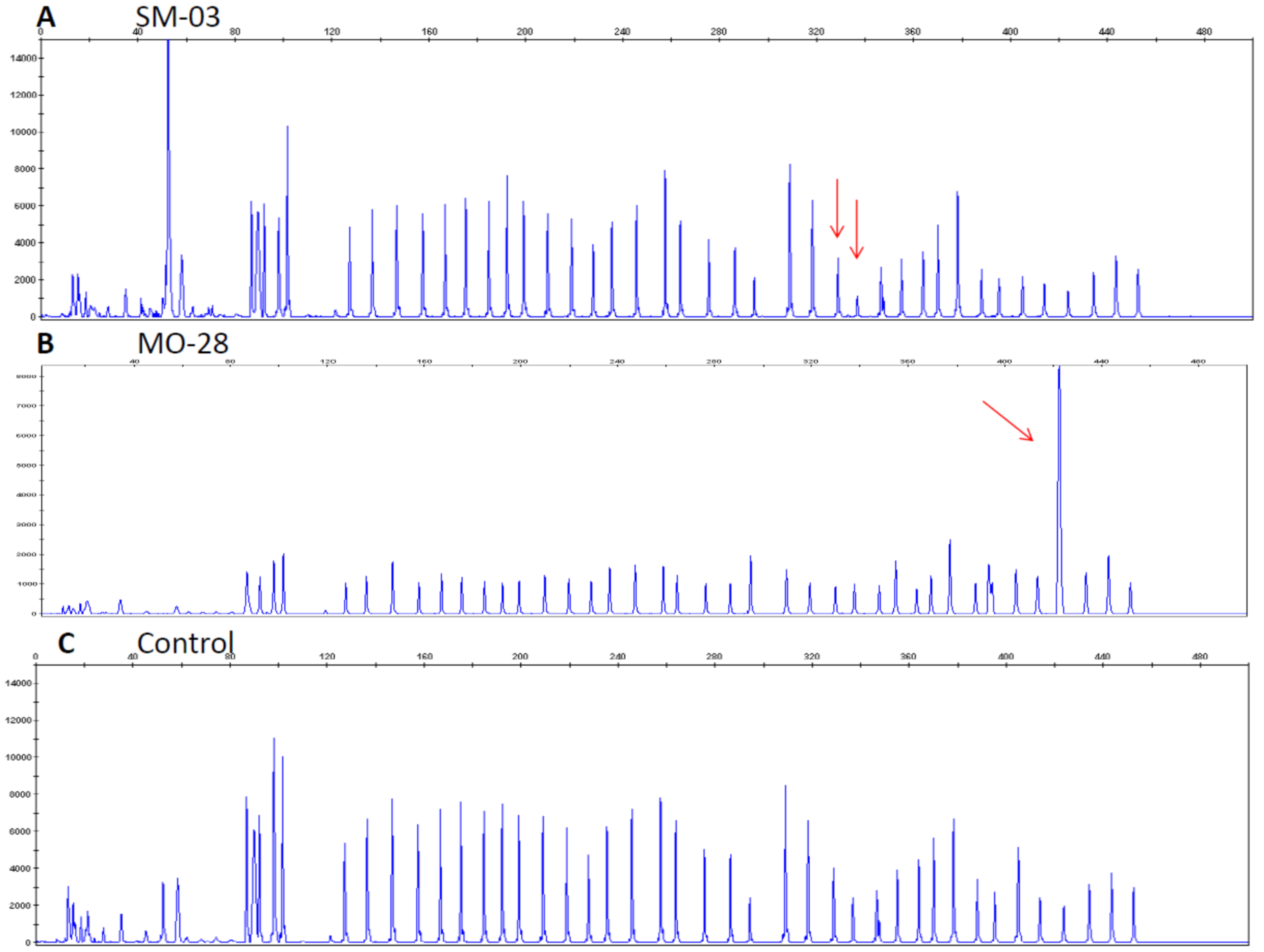

Supplement: Additional file 2 — MLPA analysis. A. Electropherograms showing reduced peaks (arrows) of exons 16 and 17 of the BRCA1 gene in patient SM-03 compared with a control sample (C), characterizing a two-exon deletion. B. Electropherogram obtained from the patient MO-28 showing the amplification (off-scale peak) of exon 24 in the BRCA1 gene (arrow). [file 1471-2350-15-55-S2.tiff]
